# The association between working memory and mathematical problem solving: A three-level meta-analysis

**DOI:** 10.3389/fpsyg.2023.1091126

**Published:** 2023-03-28

**Authors:** Zhongtian Ji, Kan Guo

**Affiliations:** School of Mathematical Sciences, Beijing Normal University, Beijing, China

**Keywords:** working memory, mathematical problem solving, executive functions, word problem, three-level meta-analysis

## Abstract

Although working memory (WM) is an important factor in mathematical problem solving (MPS), it remains unclear how well WM relates to MPS. Thus, we aimed to determine this relationship by using a meta-analysis. We searched electronic databases for studies published between 2000 and 2020 and established operational criteria. We conducted Egger’s regression tests and created funnel plots to test for publication bias. Finally, a three-level meta-analytic model analysis of data from 130 studies involving 43,938 participants and 1,355 effect sizes revealed a moderate relationship between WM and MPS (*r* = 0.280, 95% CI = [0.263, 0.314]). Moreover, moderator analyses showed that: (1) dressed-up word problems were more strongly tied to WM than to intra-mathematical problems; (2) the central executive function showed the strongest relation with MPS, whereas the phonological loop had the weakest; (3) gender ratio had significant moderating effects; and (4) some of the above-mentioned significant moderating effects were unique after controlling for other factors. Implications for research and practice were also discussed.

## 1. Introduction

Problem solving has, for some time, occupied a prominent position in education research (e.g., Lawson, 2003; Felmer et al., 2016; Priemer et al., 2020). In recent years, incorporating problem solving in school mathematics instruction has become a major area of interest within the field of mathematics education (e.g., Jitendra et al., 2005; Popham et al., 2020). From the perspective of teaching, research on the cognitive level has not been properly transferred to pedagogical issues, and remains separate from practice. Focusing on cognitive training is likely to aid in far-transferring students’ performance. However, previous studies have failed to show any stable evidence or provide impetus for teachers’ practice. Research on cognitive abilities and mathematical performance thus far has provided ideas for further exploration, and it is possible to identify the predictors for problem solving.

Working memory (WM) is frequently mentioned with regards to cognitive abilities in mathematics. Extensive studies have established that WM is related to students’ mathematical performance (e.g., Meyer et al., 2010; Ching, 2017; Wu et al., 2017; Korhonen et al., 2018; Fuchs et al., 2020). In previous studies from Baddeley and Hitch (1974) and Baddeley (1992, 2003, 2010), WM referred to a system to provide temporary storage and manipulation of the information necessary while performing complex tasks. As for mathematical problem solving (MPS), memory systems also appear to be decisive factors (Ambrus and Barczi-Veres, 2016). However, studies have yielded mixed results. For instance, Peng et al. (2016) estimated the average correlation between WM and mathematical word problem solving skills to be 0.37 while Song et al. (2011) found that students with lower WM capacity performed better on medium difficult problems than students with higher WM capacity. Solving mathematical problems appears to be complex as the process involves considerable phases (Pongsakdi et al., 2020). For mathematics, more attention should be given to “knowledge.” However, the same “knowledge” might involve different problem solving strategies and cognitive processes. For example, to calculate 8+5, a child who uses a retrieval strategy might solve it by recalling from memory, but a child who uses a decomposition strategy might break it down to 8 + (2 + 3). These are obviously different and the individual differences in memory abilities are related to individual differences in MPS. The relationship between WM and mathematics has been examined by meta-analysis (e.g., Peng et al., 2016). Although the classification divides mathematical skills (e.g., basic number knowledge, whole-number calculations, fractions, Peng et al., 2016), focusing on cognitive processes and MPS, the results are complex. For example, for geometry problem solving tasks from a standardized geometrical achievement test (Mammarella et al., 2012), children were required to calculate the area of complex figures and solve complex geometrical problems. Both calculation skills and geometry knowledge are necessary. Overall, MPS emphasizes students’ cognitive processes, providing better future direction.

In MPS, the process comprises several phases that are not necessarily performed sequentially: (1) understanding the problem information and situation; (2) translating problems into a mathematical model; (3) solving the mathematical model with mathematical skills; (4) interpreting and examining results with respect to the problem situation; and (5) communicating the results of the original problems (Pongsakdi et al., 2020). The integrity and overlapping of these processes make them difficult to break down. However, different kinds of problems might cause students varying degrees of cognitive load making it difficult to interpret students’ cognitive processes (e.g., Ayres, 2001; Jäder et al., 2017; Voica et al., 2020). In the studies of Blum et al. (2007), mathematical problems were categorized into three main types with varying focuses on cognitive processes: intra-mathematical problems, dressed-up word problems, and modeling problems. Schukajlow et al. (2012) described an intra-mathematical problem as a problem without any connection to the real world. The beginning of the cognitive process follows the mathematical model directly. Problems that appeared the most in class were dressed-up word problems (e.g., Blum, 2015). The cognitive activities involved are more complex than when solving intra-mathematical problems since the mathematical models have been dressed- up according to real-life situations. In solving modeling problems, there always exists a modeling loop and students are required to go back and forth between reality and mathematics (e.g., Herbst, 2019). Taken together, according to students’ performance on different problems, it helps to speculate and understand how cognitive factors, including WM, relate to the performance of MPS.

Furthermore, several studies have highlighted that WM relates to mathematics with the strength of these relations differing across components of WM (e.g., Costa et al., 2011; Rennie et al., 2014; Bullen et al., 2020). With their focus on knowledge and skills, previous studies do not offer an adequate explanation for the meaning between those components and the cognitive process during MPS. This article provides valuable insight for understanding the association. For instance, the central executive function might be more vital for dressed-up problems and modeling problems because students must identify what information is useful for solving the problem, plan how to apply what they know comprehensively in real life, and make decisions on how to manage the information. In solving intra-mathematical problems, the phonological loop may play a more important role in phonological awareness and coding in counting (e.g., equations, problems about a sequence of numbers). Therefore understanding the relationship between the three components and MPS is of practical significance, and will provide a fresh angle for educators to re-examine the cognitive processes in MPS. Moreover, researchers have operationalized WM and these components in a variety of ways (e.g., operation span, block span, sentence span). Previous studies have investigated the difference between MPS and WM measures based on reading and counting (Perlow and Jattuso, 2018). Clarifying these problems will also be beneficial for our understanding of the nature of MPS.

To address educators’ concerns, it is hoped that this research will contribute to a deeper understanding of students’ characteristics (e.g., grade level, gender ratio). For example, because of the changing focus of math instruction (i.e., a heavier focus on counting/calculations in primary school and at young ages, and on complex problems when the student reaches middle or high school), the role of WM may also evolve. In terms of the difficulty of the problems mentioned earlier, even when distinguished in one study, they are not comparable on a wide scale across studies. This research sought to remedy this issue by analyzing MPS by focusing more on cognitive processes rather than on the difficulty of knowledge. It is not certain whether intra-mathematical problems are easier or more difficult than dressed-up problems in different school periods. By exploring problems or cognitive processes, the results will be more general without the limitations of age or other sample types.

In summary, although studies have focused on mathematics, research has yet to systematically investigate MPS. Most studies are limited in that they may be generalizable only to mathematics knowledge, but MPS differs from mathematics in a number of important ways (e.g., understanding, comprehension, and monitoring). The aim of this study was to develop a better understanding of MPS. This paper is structured as follows: We first focus on problems that stress cognitive processes in MPS. We then discuss the components and measures of WM. This is followed by a discussion on the relationship between WM and MPS. We then explore the influence of sample type which is independent of knowledge leading to purer results. Besides, a challenge for this research is that one study may involve two types of mathematical problems or several subsystems of WM and is therefore necessary to extract more than one effect size for the same study. Common methods such as selecting only one effect size per study used in the traditional meta-analysis are unlikely to be appropriate for this study. However, by the three-level meta-analysis, all the useful effect sizes could be extracted and the heterogeneity of within-study variance was calculated to ensure independency (Ran et al., 2022). The three-level meta-analysis model has been proven to be as effective to estimate the parameters in meta-analysis as other traditional random effects approaches, with the additional advantage that multilevel models are more flexible (Van den Noortgate and Onghena, 2003; Ran et al., 2022). For example, multiple predictors can be incorporated into this model (Fernández-Castilla et al., 2020). No previous study has used the three-level meta-analysis model for analyzing the association between WM and MPS. To conclude, we answer the following two questions:

(1) What is the size of the relationship between WM and MPS?
(2) Does the relationship between WM and MPS vary as a function of (a) task type or (b) participant characteristics?

## 2. Methods

### 2.1. Data collection

**FIGURE 1:**
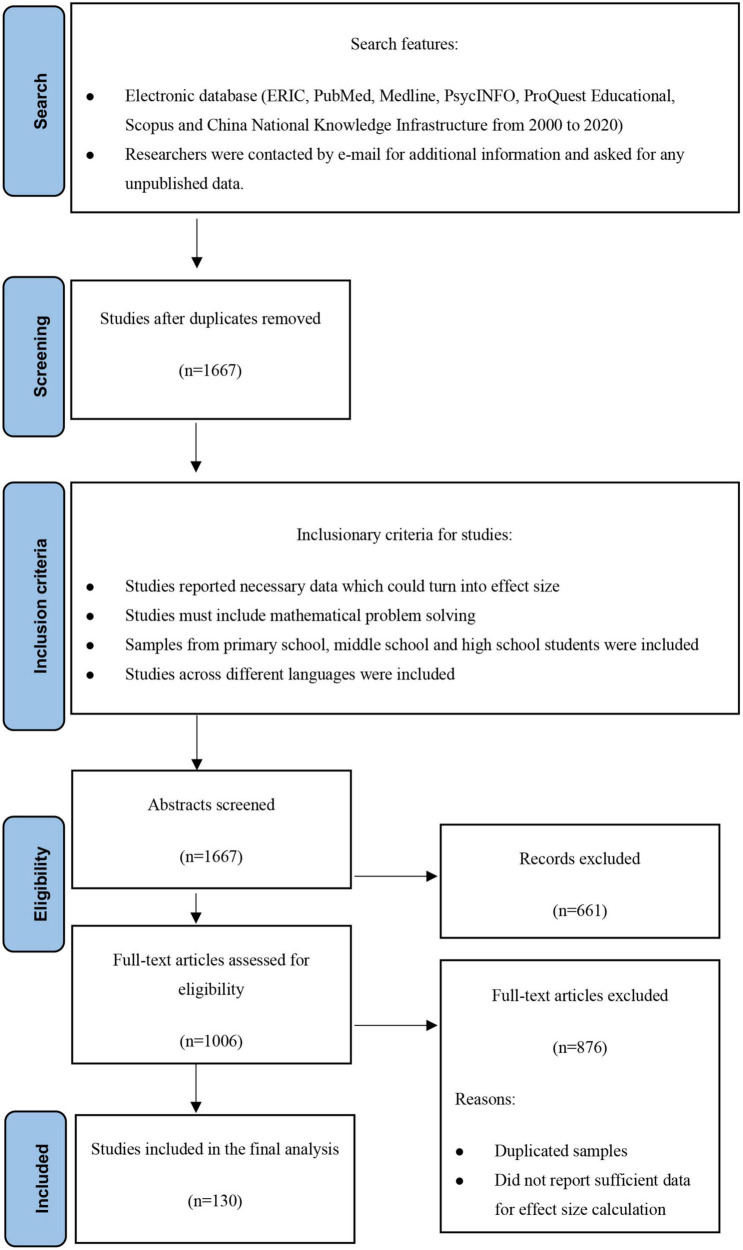
Flow diagram for search and inclusion on studies.

Figure 1 outlines the inclusion, search, and coding procedures. To identify studies for the three-level meta-analysis, we first searched electronic databases (i.e., ERIC, PubMed, Medline, PsycINFO, ProQuest Educational, Scopus, and the China National Knowledge Infrastructure) for studies published between 2000 and 2020. We used problem solving, math,* and working memory in our search, as well as the AND command. We removed the duplicates at first and contacted authors who published studies that we could not find and asked for their papers or unpublished data.

### 2.2. Operational criteria for inclusion and the elimination of studies

For the target topics included in this study, we first established operational criteria to determine the indicators. We included studies that had considered WM as a whole model or measured one of the WM components (e.g., the central executive function).

Regarding MPS, we considered two kinds of outcomes (intra-mathematical problems and dressed-up word problems). As the included research focused more on young students, there was no research using modeling problems to investigate the relationship. To be considered an intra-mathematical problem, the task had to include arithmetic problems or natural operations with no relationship to reality (e.g., addition and subtraction). To be considered a dressed-up word problem, the task had to include mathematical reasoning, applied math problems, and some necessary problem situations.

Furthermore, if a dissertation was published as an article, we only considered the article itself. After applying these criteria, we identified 130 studies with sample sizes ranging from 20 to 5,234.

### 2.3. Coding procedures

We created an online coding form. Two researchers coded the following content of the selected studies separately: (a) participant characteristics (e.g., sample size, grade level, country/region, typically developing students vs. students with difficulties); (b) type of MPS outcome (intra-mathematical problem/dressed up word problem); (c) type of WM (unspecified WM/central executive/visuospatial sketchpad/phonological loop); (d) tool for measuring WM (inventory/operation/block/sentence/digit/spot/others).

All effect sizes were then coded. Several studies have reported more than one measure to examine the relationship between WM and MPS. According to the basic rules of the three-level meta-analysis, all relevant effect sizes from each selected study were coded without reducing the number of effect sizes in any way. To ensure the robust reliability of this study, two independent recorders double-coded all the primary studies and checked the data to ensure coding accuracy. The consensus rate (Cohen’s kappa) varied between 95 and 100%. Most differences in coding were because of the lack of effective and comprehensive information provided in several studies that described the samples and measures. After revisiting the studies, and discussing the differences, two independent recorders reached an agreement (see Supplementary Appendix A).

### 2.4. Moderator variables

In each study, both groups of important moderators were coded that might explain the significance of the residual within- and between-study variance.

#### 2.4.1. Task type

We classified the WM tasks into the central executive, the visuospatial sketchpad, the phonological loop, and unspecified WM, which were unspecified in primary studies. We coded the measurement of WM to determine whether the overall effect size varied across tools. As highlighted earlier, the classification of WM tests was determined on the basis of the surface-level elements of test content. (e.g., inventory, operation, block, sentence, digit, and spot). We categorized MPS tasks into intra-mathematical and dressed-up word problems.

#### 2.4.2. Participant characteristics

We included four participant characteristics in this study. First, we coded gender based on the ratio of males included in the samples. Second, we categorized the grade levels as follows: elementary, middle, and high school. Third, we coded the cultural environment as a category variable (1 = Eastern, 0 = Western) according to the students’ country or region reported in the study (Ran et al., 2022). Finally, we coded whether the sample included students with difficulties (e.g., math difficulties and dyslexia).

### 2.5. Statistical analysis

We used the metafor package for the R statistical program (Viechtbauer, 2010) and the three-level meta-analytic model tutorial (Assink and Wibbelink, 2016) for the analysis. Since three-level models assume a normal distribution of effect sizes (Van den Noortgate et al., 2013), it is necessary to transform all data into Fisher’s *Z*-values. We applied the Fisher Z-transformation first to conduct the meta-analysis, and then Fisher’s z-values were converted back, respectively into correlation coefficients for interpretability (Hedges and Olkin, 2014; Card, 2015). Pearson’s r value was not provided in almost 20 of the 130 studies. However, using alternative formulas from, among other sources, Lenhard and Lenhard (2016), we were able to compute the r correlation and then transformed it into Fisher’s Z-score. For instance, we used a procedure for converting standardized β to r and then r could be used directly as an effect size. We were also able to compute Cohen’s d value using the statistic about means, standard deviations and sample sizes from the treatment and control group, and the t-statistic from the group test and then transformed d into the r-value (Borenstein et al., 2009). As noted in the introduction, we used a three-level random effects model and included all effect sizes in the same study. The three-level meta-analytic model considers three variance components distributed across the model’s three levels: sampling variance of the extracted effect sizes at Level 1, variance between effect sizes from the same study at Level 2, and variance between studies at Level 3 (Assink and Wibbelink, 2016).

To establish whether the variation in the *r*-value between the studies was significant, we used the *Q* test of homogeneity (Hedges and Olkin, 2014). We also computed the 95% CI for each overall effect size to provide more information regarding the correlation. We calculated the variance at Level 1 according to Cheung’s (2014) formula and applied the log-likelihood-ratio test to examine heterogeneity at levels 2 and 3. Furthermore, we tested for significance and calculated the distribution of the overall variance.

We also explored moderator variables as potential sources of additional variance in the effect size. We used linear models to predict the study’s outcome from the moderator variables, both for the continuous (i.e., gender ratio) and categorical (i.e., school level, task type, and sample characteristics) moderators. Universal classifications were chosen as the reference category to clarify the findings between different task types (e.g., unspecified working memory in WM tasks, both in MPS tasks and others in WM tests). Furthermore, we used a multiple moderator model to scrutinize the unique effect of significant moderators in the univariate analyses and added all significant moderators to the model. We tested the degree of difference between the subsets of studies using a *Q* test and by comparing the correlation magnitude with CIs between the study subsets. Similarly, we investigated the variances at levels 2 and 3.

### 2.6. Publication bias

To test for publication bias, we first conducted Egger’s regression tests (Egger et al., 1997) to test the relationship between the size of the effects from each study and the associated standard error (Georgiou et al., 2020; Ran et al., 2022). If the results of the linear regression showed no significant difference, there was no publication bias. Furthermore, we created funnel plots to test for publication bias. In the funnel plot, the standard error was plotted on the y-axis and the effect size on the x-axis, and if publication bias exists, the funnel would not be symmetric (Ludwig et al., 2019; Borenstein et al., 2021).

## 3. Results

### 3.1. Study features

Of the 130 publications included in our final analysis, 24 reported results on all three components of WM, and 55 reported results on both MPS outcomes. There were 43,938 students represented, with sample sizes ranging from 20 to 5,234. Moreover, one study exclusively focused on female students. The number of effect sizes in each study ranged from 1 to 36.

### 3.2. Meta-analytic results

The three-level meta-analytic model demonstrated that the overall mean correlations between WM and MPS were significant (*r* = 0.280, *p* < 0.001, 95% CI = [0.263, 0.314]). Additionally, the log-likelihood-ratio test showed significant heterogeneity (*p* < 0.001) at the within-study variance (Level 2) and the between-study variance. Exactly 16.34% of the total variance could be attributed to variance at Level 1, 26.05% of the total variance could be attributed to the differences between the effect sizes within studies at Level 2, and 57.61% could be attributed to the between-study variance (Level 3).

### 3.3. Results of the moderator analyses

First, we delved into the role of the three components of WM, two types of MPS tasks, and the measured elements (WM) in the relationship of interest. As outlined in Table 1, they were all significant moderators. Studies testing the central executive produced significantly larger correlations than those that tested the other two components (0.303 > 0.265 > 0.248, *p* < 0.001). Compared with other tasks, studies using intra-mathematical problems only generated a significantly smaller correlation than those using dressed-up word problems (0.309 > 0.259, *p* < 0.001). Besides, measuring WM by operation showed a larger relation than any other WM tests (*p* < 0.001).

**TABLE 1:** Relation between working memory (WM) and task types.

Moderator variable	*k*	Intercept/mean z (95% CI)	β (95% CI)	Mean *r*	*F*(df1, df2)	*p*-value	Level 2 variance	Level 3 variance
**a. Variable**								
Unspecified working memory (RC)	465	0.301*** (0.271, 0.332)		0.292	*F*(3,1353) = 7.384	<0.001***	0.008***	0.018**
Central executive	341	0.313*** (0.278, 0.347)	0.011 (−0.024, 0.047)	0.303				
Phonological loop	303	0.253*** (0.222, 0.285)	−0.048** (−0.079, −0.016)	0.248				
Visuo-spatial sketchpad	248	0.271*** (0.238, 0.303)	−0.031 (−0.063, 0.002)	0.265				
**b. Mathematical problem solving tasks**								
Both (RC)	70	0.343*** (0.290, 0.396)		0.330	*F*(2,277) = 17.360	<0.001***	0.008***	0.020***
Intra-mathematical problem	912	0.265*** (0.237, 0.293)	−0.078** (−0.127, −0.029)	0.259				
Dressed-up word problem	297	0.319*** (0.289, 0.350)	−0.024 (−0.075, 0.027)	0.309				
**c. Measured element (WM)**								
Others (RC)	608	0.285*** (0.256, 0.313)		0.278	*F*(6,274) = 4.335	<0.001***	0.009***	0.019***
Operation	22	0.410*** (0.329, 0.491)	0.125** (0.045, 0.205)	0.388				
Block	175	0.280*** (0.245, 0.315)	−0.005 (−0.034, 0.024)	0.273				
Sentence	97	0.298*** (0.259, 0.337)	0.014 (−0.020, 0.047)	0.289				
Digit	277	0.274*** (0.243, 0.305)	−0.010 (−0.035, 0.014)	0.267				
Spot	129	0.342*** (0.304, 0.380)	0.058*** (0.025, 0.090)	0.329				
Inventory	3	0.250 (−0.057, 0.557)	−0.035 (−0.343, 0.274)	0.245				

*k* = numbers of correlations; mean *z* = mean effect size (Fisher’s *z*); β = estimated regression coefficient; *r* = correlation size (Pearson’s *r*) for studies belonging to different categories of the moderator variable; Level 2 variance = variance between effect sizes extracted from the same study; Level 3 variance = variance between studies; RC = reference category. **p < 0.01; ***p < 0.001.

Second, we further analyzed the moderating effects of participant characteristics, including gender, school level, culture, and sample characteristics. As seen in Table 2, the gender ratio was a significant moderator. The regression coefficient was positive (β = 0.016), implying that this association was stronger in boys. Besides, the correlation between WM and MPS was stable across school level, cultural background, and unfolding situation.

**TABLE 2:** Relation between working memory (WM) and participant characteristics.

Moderator variable	*k*	Intercept/mean z (95% CI)	β (95% CI)	Mean *r*	*F*(df1, df2)	*p-*value	Level 2 variance	Level 3 variance
a. Gender	1063	0.260*** (0.232, 0.288)	0.016*** (0.009, 0.024)	0.254	*F*(1,1061) = 16.639	<0.001***	0.007***	0.015***
b. School level								
Primary school (RC)	1188	0.280*** (0.255, 306)		0.273	*F*(2,1236) = 0.533	0.587	0.009***	0.015***
Middle school	36	0.281*** (0.144, 0.418)	0.001 (−0.138, 0.140)	0.274				
High school	25	0.364*** (0.207, 0.521)	0.084 (−0.075, 0.243)	0.349				
c. Culture								
Western (RC)	1066	0.288*** (0.258, 0.317)		0.280	*F*(1,1234) = 0.023	0.881	0.009***	0.017***
Eastern	170	0.283*** (0.225, 0.341)	−0.005 (−0.070, 0.060)	0.276				
d. Sample characteristics								
Typically-developing students (RC)	1050	0.286*** (0.258, 0.313)		0.278	*F*(1,1355) = 0.348	0.556	0.008***	0.018***
Partly children with difficulties	87	0.300*** (0.253, 0.348)	0.015 (−0.034, 0.063)	0.291				

k = numbers of correlations; mean z = mean effect size (Fisher’s z); β = estimated regression coefficient; r = correlation size (Pearson’s r) for studies belonging to different categories of the moderator variable; Level 2 variance = variance between effect sizes extracted from the same study; Level 3 variance = variance between studies; RC = reference category. ***p < 0.001.

Previous studies have demonstrated that moderators might be interrelated (Hox et al., 2017). Therefore, we added all the significant moderators to the multiple moderator model to examine what effects were really relevant. As mentioned earlier, we chose universal classifications as the reference category. The omnibus test showed significant results, *F*(12,973) = 7.676, *p* < 0.001, suggesting that at least one of the regression coefficients of the moderators significantly deviated from zero. Based on the findings in Table 3, we were able to assert that the components of WM, MPS tasks and measured elements for WM were not confounded by the gender ratio. These results indicated these three moderators had a uniquely moderating effect on the association.

**TABLE 3:** Multiple moderator model on the relation between working memory (WM) and mathematical problem solving (MPS).

Moderator variable			*k*	β (95% CI)	
	Intercept			0.428(0.332, 0.524)***	
a. Gender ratio				−0.057(−0.125, 0.010)	
b. Components of working memory	Central executive		243	−0.003(−0.045, 0.038)	
	Phonological loop		203	−0.020(−0.062, 0.022)	
	Visuo-spatial sketchpad		214	−0.061(−0.119, −0.003)*	
c. Mathematical problem solving tasks	Intra-mathematical problem		691	−0.050(0.015, 0.084)**	
	Dressed up word problem		242	−0.026(−0.125, 0.073)	
c. Measured element (WM)	Operation		13	0.102(0.012, 0.191)*	
	Block		147	−0.033(−0.072, 0.005)	
	Sentence		91	0.006(−0.027, 0.039)	
	Digit		200	−0.029(−0.057, −0.002)*	
	Spot		97	0.025(−0.011, 0.061)	
	Inventory		3	−0.064(−0.331, 0.203)	
Multiple moderator model	*k* = 986	*F*(12,973) = 7.676	*p* < 0.001	Level 2 0.007***	Level 3 0.013***

*k* = numbers of correlations; β = estimated regression coefficient; Level 2 = variance between effect sizes extracted from the same study; Level 3 = variance between studies. *p < 0.05; **p < 0.01; ***p < 0.001.

### 3.4. Publication bias

**FIGURE 2:**
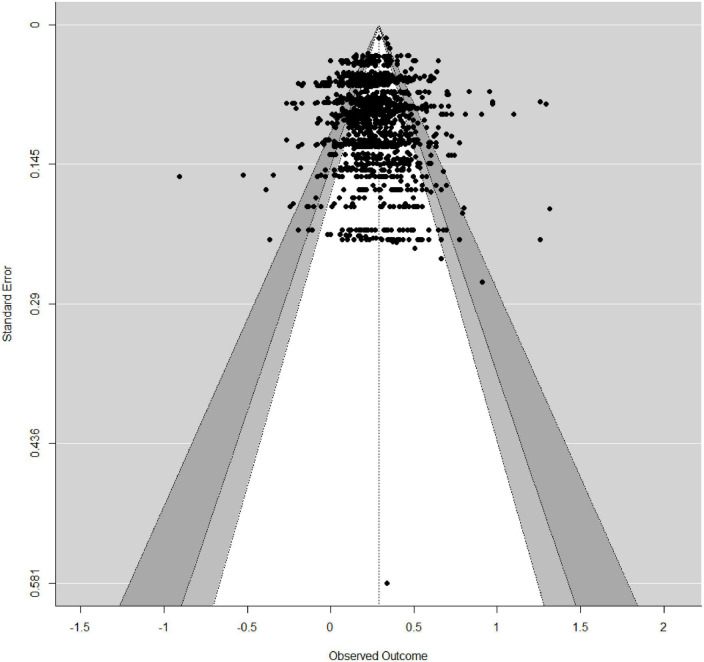
Funnel plot of the overall mean r analysis.

The results of Egger’s test suggested that publication bias should be ignored in the meta-analysis because the *p*-value of this test exceeded 0.05. The symmetric distribution of the funnel plot was depicted in Figure 2, which indicated that the results of our meta-analysis were stable and reliable.

## 4. Discussion

The purpose of this meta-analysis was to estimate the size of the relationship between WM and MPS and to determine if different factors (task type/participant characteristics) moderate their relationship. When we considered any correlation between any components of WM, we found significant links between WM and MPS (the average correlation was 0.280), which are similar to those reported in previous meta-analyses (Peng et al., 2016). Additionally, this relationship was significantly influenced by publication characteristics and task type, but not by participant characteristics. Concretely speaking, in terms of WM, all components of WM demonstrated significant ties with MPS, and the central executive showed the strongest relationship (*r* = 0.303). Regarding the WM tests, operation span had a strong relationship with MPS. In the field of outcomes for MPS, the connection between WM and dressed-up word problems was stronger. Furthermore, although gender ratio had significance, the results of the multiple moderator model indicated that it was not as steady as expected.

In MPS, diverse strategies and cognitive processes were identified as different types of math problems (Star and Rittle-Johnson, 2008; Hu et al., 2017; Rott et al., 2021). Since different types of problems require different cognitive abilities, we investigated this by examining the relationship between WM and intra-mathematical or dressed-up word problems (Rellensmann and Schukajlow, 2017; Krawitz and Schukajlow, 2018). In this meta-analysis, intra-mathematical problems appeared to be purely mathematical tasks and could not lead to any reality-related mental activities. In contrast, dressed-up word problems are more common in daily life and may require more cognitive resources. Students may have to identify missing or useful information, “undress” the problems, and experience the process of mathematization.

In this meta-analysis, we found that two kinds of mathematical problems were both positively related to WM and the types of MPS indeed moderated the relationship. Furthermore, dressed-up word problems showed stronger links than intra-mathematical problems (*r* = 0.309 for dressed-up word problems). Additionally, the results of the multiple moderator model indicated similar results. Taken together, these findings imply that cognitive processes drive the relationship between WM and MPS, and highlight their important roles. Understanding the problem situations and translating them into a mathematical model might draw upon significant WM resources. Currently, problem solving is no longer thought of as solving pure mathematical problems (Holmes et al., 2017; Priemer et al., 2020). Recent developments in science, technology, engineering, and mathematics (STEM) and project-based learning place a strong emphasis on 21st-century skills, such as solving problems in reality (Markham et al., 2003; Chen and Yang, 2019; Priemer et al., 2020). As mathematical educators, we also expect students to apply math to real-life scenarios. Students are required to practice solving more reality-related problems, such as ill-structured problems (e.g., Jäder et al., 2017), rather than intra-mathematical problems, thus they also need more regulation of cognition and experience more complex processes. Based on such a trend, WM may play a larger role in identifying valuable information, organizing, and monitoring total performance, and rethinking outcomes in real life.

Regarding WM, we found that the relationship between MPS and WM is indeed affected by components of WM, and the central executive function indicated the strongest relationship with MPS, whereas the phonological loop had the weakest relationship (*r* = 0.303 for the central executive, *r* = 0.248 for the phonological loop, and *r* = 0.265 for the visuospatial sketchpad). Given the variety of WM tests, we also found that each WM test-MPS relationship was well documented and the tests measuring operations showed stronger links than other types. Previous studies have demonstrated that WM tests measure both cognitive abilities and other skills (e.g., counting numbers) according to how the construct was conducted and assessors always use the tool relating best to their criterion of interest (Perlow and Jattuso, 2018). However, the results show that practitioners should pay more attention to cognitive characteristics or relationship between WM tests and other constructs. Besides, several cognitive processes of the executive system might influence WM in MPS, such as controlling, encoding, and retrieval strategies, and suppressing unnecessary information (Miyake et al., 2000; Oberauer et al., 2003). Multiple moderator model analyses also proved that the central executive function showed a stronger relationship than the other two components. Taken together, students need to better master how to organize the entire MPS process. To promote students’ performance in MPS, training the central executive function would be a powerful strategy. As mentioned earlier, because problem solving integrates information from different branches of math, students may need to have a solid command of switching strategies flexibly, and the relationship between WM and MPS should be invariant.

Although gender ratio showed significance, its moderating effects were not robust after controlling for other factors. School level and other participant characteristics did not moderate the relationship between WM and MPS either. Rooted in the idea that cognitive universals exist in this relationship, we claimed that the contributions made here have wide applicability. Additionally, although the school level had a non-significant moderation effect, students at higher school levels showed a larger relationship (0.349 > 0.274 > 0.273). Young students always rely on some basic problem solving strategies such as finger counting to solve mathematical problems at the beginning of formal schooling (Ramirez et al., 2016; Palmer and van Bommel, 2020). By practicing these strategies, they develop strong problem–answer associations. Thus, when they grow up and use more advanced problem solving strategies, they rely heavily on memory-based processes (Siegler and Shrager, 1984; Laski et al., 2013). Another possible reason is due to the different developing patterns among the three components of WM. Studies focusing on working memory have found that the central executive matures later (Palmer, 2000; Muñoz-Pradas et al., 2021), which provides a plausible explanation for our finding. However, the current research still pays insufficient attention to the relationship between WM and MPS in high school students. We recommend additional research to focus on the relationship in senior grades, and apply lab-based findings to actual situations (Cui and Guo, 2022). Clearly, our meta-analysis synthesized research from multiple sources and obtained relatively more reliable conclusions than a single study, thus to some extent making up for the current deficiencies. A greater focus on that could produce interesting findings that develop a deeper understanding of the relationships between WM and MPS.

Our study has some limitations. Although we searched for unpublished papers, this might be a problem for the current study, with a bias toward significant effects. Thus, although funnel plot analyses and Egger’s test confirmed that publication bias was probably not a major problem in this study, we may have missed studies that reported non-significant results. Besides, we acknowledge that some of the categories in the moderator analyses did not include many studies. For instance, when investigating the role of school level/grade in the relationship, we had only three students in high school and four in middle school. This may have influenced the chances of finding significant differences. Third, we did not examine the relationship between problem solving and WM. The reason for this circumstance is that primary studies rarely report on the relationship between WM and each process, such as identifying information. Hence, we strongly recommend that future empirical research about this relationship pay more attention to the specific process of MPS. This would allow researchers to further explain how WM is related to MPS. Finally, we did not control for the role of instruction in the relationship between WM and MPS. Previous studies indicate that students’ MPS competency can be improved through training and practice (Witt, 2011). Different forms of instruction can alter the cognitive processes involved in specific problems.

In summary, the present meta-analysis applied a three-level, meta-analytic model to quantitatively synthesize the overall association between WM and MPS. The manuscript, therefore, adds to a growing body of research on the role of WM (e.g., Reber and Kotovsky, 1997; Justicia-Galiano et al., 2017). Therefore, all evidence supports the significantly positive correlation between WM and MPS, suggesting that there are benefits if we develop students’ WM abilities, which are linked to mathematical performance. Subsequent moderator analyses demonstrated some significant moderators that could explain differences in the strength of the relationship, namely publication characteristics and the task type of WM, as well as MPS. These results have direct implications for instruction and interventions in programming. However, this meta-analysis also underscores areas for future research, including processes of MPS and specific populations [e.g., students with math-related disabilities, which may have significant benefits in terms of mathematical cognition (Geary et al., 2005)].

## Data availability statement

The original contributions presented in this study are included in the article/Supplementary material, further inquiries can be directed to the corresponding author.

## Author contributions

KG collected and organized the data and critically reviewed and revised the manuscript. ZJ performed the statistical analysis. KG and ZJ wrote the original draft. Both authors contributed to the manuscript revision and approved the submitted version.
