# Supplementary material for: The association between working memory and mathematical problem solving: A three-level meta-analysis
Source: Front Psychol. 2023 Mar 28;14:1091126. doi: 10.3389/fpsyg.2023.1091126 (PMC10086603; doi:10.3389/fpsyg.2023.1091126)
Supplement: Supplementary file 1 [file Table_1.DOCX]

Supplementary Material

# Appendix A

| Study | Gender ratio | Sample characteristic^a^ | School level | Culture background | MPS task^a^ | Element for WM | Components for WM^b^ | Fisher' *z* |
| --- | --- | --- | --- | --- | --- | --- | --- | --- |
| Bahar& Maker (2015) | 1.16129 | 0 | primary | western | both | others | UWM | 0.266108 |
|  | 1.16129 | 1 | primary | western | both | others | UWM | -0.090244 |
| Bullen et al. (2020) |  | 1 |  | western | both | others | UWM | 0.459897 |
|  |  | 1 |  | western | both | others | PL | 0.423649 |
|  |  | 1 |  | western | both | others | UWM | 0.725005 |
|  |  | 1 |  | western | both | others | UWM | 0.549306 |
|  |  | 1 |  | western | both | others | PL | 0.320545 |
|  |  | 1 |  | western | both | others | UWM | 0.693147 |
|  |  | 1 |  | western | both | others | UWM | -0.080171 |
|  |  | 1 |  | western | both | others | PL | 0.070115 |
|  |  | 1 |  | western | both | others | UWM | 0.549306 |
|  |  | 1 |  | western | intra | others | UWM | 0.234189 |
|  |  | 1 |  | western | intra | others | PL | 0.266108 |
|  |  | 1 |  | western | intra | others | UWM | 0.618381 |
|  |  | 1 |  | western | intra | others | UWM | 0.459897 |
|  |  | 1 |  | western | intra | others | PL | 0.255413 |
|  |  | 1 |  | western | intra | others | UWM | 0.632833 |
|  |  | 1 |  | western | intra | others | UWM | -0.030009 |
|  |  | 1 |  | western | intra | others | PL | 0.161387 |
|  |  | 1 |  | western | intra | others | UWM | 0.376886 |
| Costa et al. (2011) |  | 1 |  | western | both | digit | PL | 0.161387 |
|  |  | 1 |  | western | dressed | digit | PL | 0.161387 |
|  |  | 1 |  | western | intra | digit | PL | 0.202733 |
|  |  | 1 |  | western | intra | digit | PL | 0.120581 |
|  |  | 1 |  | western | intra | digit | PL | 0.234189 |
|  |  | 1 |  | western | intra | digit | PL | 0.01 |
|  |  | 1 |  | western | both | digit | PL | 0.223656 |
|  |  | 1 |  | western | dressed | digit | PL | 0.192337 |
|  |  | 1 |  | western | intra | digit | PL | 0.110447 |
|  |  | 1 |  | western | intra | digit | PL | 0.161387 |
|  |  | 1 |  | western | intra | digit | PL | 0.192337 |
|  |  | 1 |  | western | intra | digit | PL | -0.020003 |
|  |  | 1 |  | western | both | block | VS | 0.320545 |
|  |  | 1 |  | western | dressed | block | VS | 0.287682 |
|  |  | 1 |  | western | intra | block | PL | 0.365444 |
|  |  | 1 |  | western | intra | block | VS | 0.276864 |
|  |  | 1 |  | western | intra | block | VS | 0.287682 |
|  |  | 1 |  | western | intra | block | VS | 0.040021 |
|  |  | 1 |  | western | both | block | VS | 0.298566 |
|  |  | 1 |  | western | dressed | block | VS | 0.320545 |
|  |  | 1 |  | western | intra | block | PL | 0.435611 |
|  |  | 1 |  | western | intra | block | VS | 0.266108 |
|  |  | 1 |  | western | intra | block | VS | 0.30952 |
|  |  | 1 |  | western | intra | block | VS | -0.080171 |
| Rennie et al. (2014) |  | 1 | primary | western | intra | digit | UWM | 0.388423 |
|  |  | 1 | primary | western | intra | digit | UWM | 0.276864 |
|  |  | 1 | primary | western | intra | digit | UWM | 0.4118 |
|  |  | 1 | primary | western | intra | digit | UWM | 0.604156 |
|  |  | 1 | primary | western | dressed | digit | UWM | 0.435611 |
|  |  | 1 | primary | western | intra | others | CE | 0.590145 |
|  |  | 1 | primary | western | intra | others | CE | 0.472231 |
|  |  | 1 | primary | western | intra | others | CE | 0.234189 |
|  |  | 1 | primary | western | intra | others | CE | 0.365444 |
|  |  | 1 | primary | western | dressed | others | CE | 0.354093 |
|  |  | 1 | primary | western | intra | others | VS | 0.53606 |
|  |  | 1 | primary | western | intra | others | VS | 0.435611 |
|  |  | 1 | primary | western | intra | others | VS | 0.379186 |
|  |  | 1 | primary | western | intra | others | VS | 0.342828 |
|  |  | 1 | primary | western | dressed | others | VS | 0.181983 |
| HAJIBABA et al. (2013) | 0 | 0 | high | eastern | intra | digit | UWM | 0.16755 |
|  | 0 | 0 | high | eastern | intra | digit | UWM | 0.112472 |
|  | 0 | 0 | high | eastern | intra | digit | UWM | 0.097306 |
|  | 0 | 0 | high | eastern | intra | digit | UWM | 0.266108 |
|  | 0 | 0 | high | eastern | intra | digit | UWM | 0.147051 |
|  | 0 | 0 | high | eastern | intra | digit | UWM | 0.179916 |
|  | 0 | 0 | high | eastern | intra | digit | UWM | 0.224707 |
|  | 0 | 0 | high | eastern | intra | digit | UWM | 0.190263 |
|  | 0 | 0 | high | eastern | intra | digit | UWM | 0.235246 |
|  | 0 | 0 | high | eastern | intra | digit | UWM | 0.13074 |
| Aran Filippetti & Cristina Richaud (2017) | 1.185185 | 0 | primary |  | intra | digit | UWM | 0.435611 |
|  | 1.185185 | 0 | primary |  | intra | digit | UWM | 1.098612 |
|  | 1.185185 | 0 | primary |  | dressed | digit | UWM | 0.677666 |
|  | 1.185185 | 0 | primary |  | intra | others | UWM | 0.662463 |
|  | 1.185185 | 0 | primary |  | intra | others | UWM | 0.725005 |
|  | 1.185185 | 0 | primary |  | dressed | others | UWM | 0.4847 |
|  | 1.185185 | 0 | primary |  | intra | others | UWM | 0.810743 |
|  | 1.185185 | 0 | primary |  | intra | others | UWM | 0.907645 |
|  | 1.185185 | 0 | primary |  | dressed | others | UWM | 0.693147 |
| Zhu et al. (2011) | 1.179775 | 0 | primary | eastern | both | block | VS | 0.468517 |
|  | 1.179775 | 0 | primary | eastern | intra | block | VS | 0.313921 |
|  | 1.179775 | 0 | primary | eastern | dressed | block | VS | 0.375737 |
|  | 1.179775 | 0 | primary | eastern | dressed | block | VS | 0.319439 |
|  | 1.179775 | 0 | primary | eastern | both | block | VS | 0.255413 |
|  | 1.179775 | 0 | primary | eastern | intra | block | VS | 0.033012 |
|  | 1.179775 | 0 | primary | eastern | dressed | block | VS | 0.269329 |
|  | 1.179775 | 0 | primary | eastern | dressed | block | VS | 0.110447 |
|  | 1.179775 | 0 | primary | eastern | both | others | VS | 0.313921 |
|  | 1.179775 | 0 | primary | eastern | intra | others | VS | 0.164467 |
|  | 1.179775 | 0 | primary | eastern | dressed | others | VS | 0.708921 |
|  | 1.179775 | 0 | primary | eastern | dressed | others | VS | 0.699421 |
| Träff et al. (2018) | 0.675042 | 0 |  | western | dressed | block | VS | 0.342828 |
|  | 0.675042 | 0 |  | western | intra | block | VS | 0.320545 |
|  | 0.675042 | 0 |  | western | intra | block | VS | 0.298566 |
|  | 0.675042 | 0 |  | western | dressed | digit | PL | 0.331647 |
|  | 0.675042 | 0 |  | western | intra | digit | PL | 0.266108 |
|  | 0.675042 | 0 |  | western | intra | digit | PL | 0.181983 |
| Dube & Robinson (2010) | 0.975 | 0 |  | western | intra | digit | UWM | 0.161387 |
|  | 0.975 | 0 |  | western | intra | digit | UWM | 0.30952 |
|  | 0.975 | 0 |  | western | intra | others | UWM | 0.110447 |
|  | 0.975 | 0 |  | western | intra | others | UWM | 0.15114 |
|  | 0.975 | 0 |  | western | intra | digit | UWM | 0.30952 |
|  | 0.975 | 0 |  | western | intra | digit | UWM | 0.376886 |
|  | 0.975 | 0 |  | western | intra | spot | UWM | 0.331647 |
|  | 0.975 | 0 |  | western | intra | spot | UWM | 0.320545 |
| Korhonen et al. (2018) | 0.95 | 0 | primary | western |  |  | UWM | 0.192337 |
|  | 0.95 | 0 | primary | western |  |  | UWM | 0.223656 |
|  | 0.95 | 0 | primary | western |  |  | UWM | 0.110447 |
|  | 0.95 | 0 | primary | western |  |  | UWM | 0.244774 |
|  | 0.95 | 0 | primary | western |  |  | UWM | 0.192337 |
|  | 0.95 | 0 | primary | western |  |  | UWM | 0.192337 |
|  | 0.95 | 0 | primary | western |  |  | UWM | 0.181983 |
|  | 0.95 | 0 | primary | western |  |  | UWM | 0.213171 |
|  | 0.95 | 0 | primary | western |  |  | UWM | 0.100335 |
|  | 0.95 | 0 | primary | western |  |  | UWM | 0.161387 |
|  | 0.95 | 0 | primary | western |  |  | UWM | 0.140926 |
|  | 0.95 | 0 | primary | western |  |  | UWM | 0.15114 |
|  | 0.95 | 0 | primary | western |  |  | UWM | 0.223656 |
|  | 0.95 | 0 | primary | western |  |  | UWM | 0.244774 |
|  | 0.95 | 0 | primary | western |  |  | UWM | 0.15114 |
|  | 0.95 | 0 | primary | western |  |  | UWM | 0.266108 |
|  | 0.95 | 0 | primary | western |  |  | UWM | 0.213171 |
|  | 0.95 | 0 | primary | western |  |  | UWM | 0.192337 |
|  | 0.95 | 0 | primary | western |  |  | UWM | 0.15114 |
|  | 0.95 | 0 | primary | western |  |  | UWM | 0.192337 |
|  | 0.95 | 0 | primary | western |  |  | UWM | 0.070115 |
|  | 0.95 | 0 | primary | western |  |  | UWM | 0.181983 |
|  | 0.95 | 0 | primary | western |  |  | UWM | 0.140926 |
|  | 0.95 | 0 | primary | western |  |  | UWM | 0.15114 |
|  | 0.95 | 0 | primary | western |  |  | UWM | 0.202733 |
|  | 0.95 | 0 | primary | western |  |  | UWM | 0.15114 |
|  | 0.95 | 0 | primary | western |  |  | UWM | 0.15114 |
|  | 0.95 | 0 | primary | western |  |  | UWM | 0.202733 |
|  | 0.95 | 0 | primary | western |  |  | UWM | 0.234189 |
|  | 0.95 | 0 | primary | western |  |  | UWM | 0.161387 |
| Wong (2018) | 1.137931 | 0 | primary | eastern | both | digit | UWM | 0.31723 |
|  | 1.137931 | 0 | primary | eastern | dressed | digit | UWM | 0.116525 |
|  | 1.137931 | 0 | primary | eastern | intra | digit | UWM | 0.245836 |
| Li et al. (2003) | 0.864407 | 0 | primary | eastern | dressed | digit | UWM | 0.7984 |
| Friedman et al. (2018) |  | 1 | primary | western | intra | others | CE | 0.51007 |
|  |  | 1 | primary | western | dressed | others | CE | 0.590145 |
|  |  | 1 | primary | western | intra | others | PL | 0.320545 |
|  |  | 1 | primary | western | dressed | others | PL | 0.331647 |
|  |  | 1 | primary | western | intra | spot | VS | 0.287682 |
|  |  | 1 | primary | western | dressed | spot | VS | 0.354093 |
| Zhong (2009) | 1.163265 | 0 | primary | eastern | dressed | others | CE | 0.182 |
|  | 1.163265 | 0 | primary | eastern | dressed | digit | PL | 0.1614 |
|  | 1.163265 | 0 | primary | eastern | dressed | block | VS | 0.1307 |
| Lee et al. (2011) | 0.961039 | 0 | primary |  | intra | sentence | CE | 0.244774 |
|  | 0.961039 | 0 | primary |  | intra | sentence | CE | 0.223656 |
|  | 0.961039 | 0 | primary |  | intra | sentence | CE | 0.213171 |
|  | 0.961039 | 0 | primary |  | intra | sentence | CE | 0.213171 |
|  | 0.961039 | 0 | primary |  | intra | sentence | CE | 0.15114 |
|  | 0.961039 | 0 | primary |  | intra | sentence | CE | 0.234189 |
|  | 0.961039 | 0 | primary |  | intra | sentence | CE | 0.192337 |
|  | 0.961039 | 0 | primary |  | intra | sentence | CE | 0.331647 |
|  | 0.961039 | 0 | primary |  | intra | sentence | CE | 0.266108 |
|  | 0.961039 | 0 | primary |  | intra | sentence | CE | 0.276864 |
|  | 0.961039 | 0 | primary |  | intra | others | CE | 0.298566 |
|  | 0.961039 | 0 | primary |  | intra | others | CE | 0.223656 |
|  | 0.961039 | 0 | primary |  | intra | others | CE | 0.276864 |
|  | 0.961039 | 0 | primary |  | intra | others | CE | 0.447692 |
|  | 0.961039 | 0 | primary |  | intra | others | CE | 0.276864 |
|  | 0.961039 | 0 | primary |  | intra | others | CE | 0.244774 |
|  | 0.961039 | 0 | primary |  | intra | others | CE | 0.255413 |
|  | 0.961039 | 0 | primary |  | intra | others | CE | 0.365444 |
|  | 0.961039 | 0 | primary |  | intra | others | CE | 0.30952 |
|  | 0.961039 | 0 | primary |  | intra | others | CE | 0.354093 |
| Bizzaro et al. (2018) | 0.8 | 0 | primary |  | intra | digit | UWM | 0.257547 |
|  | 0.8 | 0 | primary |  | intra | digit | UWM | 0.128706 |
|  | 0.8 | 0 | primary |  | intra | digit | UWM | 0.165495 |
|  | 0.8 | 0 | primary |  | intra | digit | UWM | 0.19753 |
|  | 0.8 | 0 | primary |  | intra | digit | UWM | 0.268255 |
|  | 0.8 | 0 | primary |  | intra | digit | UWM | 0.154211 |
|  | 0.8 | 0 | primary |  | intra | others | VS | 0.236302 |
|  | 0.8 | 0 | primary |  | intra | others | VS | 0.17682 |
|  | 0.8 | 0 | primary |  | intra | others | VS | 0.03101 |
|  | 0.8 | 0 | primary |  | intra | others | VS | 0.107411 |
|  | 0.8 | 0 | primary |  | intra | others | VS | 0.366584 |
|  | 0.8 | 0 | primary |  | intra | others | UWM | 0.169608 |
| Wang et al. (2010) | 1 | 0 | primary | eastern | dressed | digit | PL | 0.3465 |
|  | 1 | 0 | primary | eastern | dressed | others | VS | 0.2224 |
|  | 1 | 0 | primary | eastern | dressed | digit | CE | 0.438 |
|  | 1 | 0 | primary | eastern | dressed | others | CE | 0.1932 |
| Jogi & Kikas (2016) | 1.096436 | 0 |  | western | intra | others | CE | 0.140926 |
|  | 1.096436 | 0 |  | western | dressed | others | CE | 0.192337 |
|  | 1.096436 | 0 |  | western | intra | others | CE | 0.15114 |
|  | 1.096436 | 0 |  | western | dressed | others | CE | 0.320545 |
| Dong (2003) | 1.137931 | 0 | primary | eastern | dressed | others | UWM | 1.3162 |
| Zebec et al. (2015) |  | 0 |  | western | intra | digit | UWM | 1.256153 |
|  |  | 0 |  | western | intra | digit | UWM | 0.972955 |
|  |  | 0 |  | western | intra | digit | UWM | 1.293345 |
|  |  | 0 |  | western | intra | digit | UWM | 0.972955 |
|  |  | 0 |  | western | intra | digit | UWM | 0.472231 |
| Zhao (2012) |  | 0 | middle | eastern | both | operation | UWM | 0.3589 |
|  |  | 0 | middle | eastern | both | operation | UWM | 0.1719 |
|  |  | 0 | middle | eastern | both | operation | UWM | 0.2711 |
|  |  | 0 | middle | eastern | both | operation | UWM | 0.0681 |
| Foley et al. (2017) | 1.108108 | 0 | primary | western | intra | others | PL | 0.4118 |
|  | 1.108108 | 0 | primary | western | intra | spot | VS | 0.244774 |
| Zhu (2013) | 1.242105 | 0 | middle | eastern | both | digit | PL | 0.4587 |
|  | 1.242105 | 0 | middle | eastern | intra | digit | PL | 0.3194 |
|  | 1.242105 | 0 | middle | eastern | dressed | digit | PL | 0.3139 |
|  | 1.242105 | 0 | middle | eastern | dressed | digit | PL | 0.3666 |
|  | 1.242105 | 0 | middle | eastern | intra | digit | PL | 0.1996 |
|  | 1.242105 | 0 | middle | eastern | both | others | VS | 0.3826 |
|  | 1.242105 | 0 | middle | eastern | intra | others | VS | 0.6994 |
|  | 1.242105 | 0 | middle | eastern | dressed | others | VS | 0.2205 |
|  | 1.242105 | 0 | middle | eastern | dressed | others | VS | 0.4177 |
|  | 1.242105 | 0 | middle | eastern | intra | others | VS | 0.209 |
|  | 1.242105 | 0 | middle | eastern | both | operation | CE | 0.9527 |
|  | 1.242105 | 0 | middle | eastern | intra | operation | CE | 0.1501 |
|  | 1.242105 | 0 | middle | eastern | dressed | operation | CE | 0.8291 |
|  | 1.242105 | 0 | middle | eastern | dressed | operation | CE | 0.5668 |
|  | 1.242105 | 0 | middle | eastern | intra | operation | CE | 0.3428 |
| Trezise & Reeve (2014) | 2.405405 | 0 |  | western | intra | operation | UWM | 0.245836 |
|  | 2.405405 | 0 |  | western | intra | operation | UWM | 0.577711 |
|  | 2.405405 | 0 |  | western | intra | block | VS | 0.267181 |
| Passolunghi et al. (2008) | 0.714286 | 0 | primary | western |  | others | UWM | 0.276864 |
|  | 0.714286 | 0 | primary | western |  | digit | UWM | 0.435611 |
|  | 0.714286 | 0 | primary | western |  | sentence | UWM | 0.472231 |
|  | 0.714286 | 0 | primary | western |  | others | UWM | 0.287682 |
|  | 0.714286 | 0 | primary | western |  | digit | UWM | 0.4118 |
|  | 0.714286 | 0 | primary | western |  | others | UWM | 0.090244 |
|  | 0.714286 | 0 | primary | western |  | digit | UWM | 0.090244 |
|  | 0.714286 | 0 | primary | western |  | sentence | UWM | 0.266108 |
|  | 0.714286 | 0 | primary | western |  | others | UWM | 0.266108 |
|  | 0.714286 | 0 | primary | western |  | digit | UWM | 0.140926 |
|  | 0.714286 | 0 | primary | western |  | others | UWM | 0.255413 |
|  | 0.714286 | 0 | primary | western |  | digit | UWM | 0.244774 |
|  | 0.714286 | 0 | primary | western |  | sentence | UWM | 0.234189 |
|  | 0.714286 | 0 | primary | western |  | others | UWM | 0.120581 |
|  | 0.714286 | 0 | primary | western |  | digit | UWM | 0.213171 |
| Villeneuve et al. (2019) |  | 0 | primary | western | intra |  | UWM | 0.080171 |
|  |  | 0 | primary | western | dressed |  | UWM | 0.110447 |
|  |  | 0 | primary | western | intra |  | UWM | 0.090244 |
|  |  | 0 | primary | western | dressed |  | UWM | 0.100335 |
|  |  | 0 |  | western | intra |  | UWM | 0.080171 |
|  |  | 0 |  | western | dressed |  | UWM | 0.090244 |
|  |  | 0 |  | western | intra |  | UWM | 0.100335 |
|  |  | 0 |  | western | dressed |  | UWM | 0.040021 |
|  |  | 0 |  | western | intra |  | UWM | 0.070115 |
|  |  | 0 |  | western | dressed |  | UWM | 0.030009 |
|  |  | 0 |  | western | intra |  | UWM | 0.100335 |
|  |  | 0 |  | western | dressed |  | UWM | 0.050042 |
| Zhang et al. (2018) | 1.109091 | 0 | primary | eastern | intra | inventory | CE | 0.13074 |
|  | 1.109091 | 0 | primary | eastern | dressed | inventory | CE | 0.320545 |
|  | 1.109091 | 0 | primary | eastern | dressed | inventory | CE | 0.298566 |
| Wang et al. (2016) | 0.886792 | 0 | primary | western | dressed | digit | CE | 0.365444 |
|  | 0.886792 | 0 | primary | western | dressed | digit | CE | 0.40006 |
|  | 0.886792 | 0 | primary | western | intra | digit | CE | 0.244774 |
|  | 0.886792 | 0 | primary | western | dressed | digit | CE | 0.497311 |
|  | 0.886792 | 0 | primary | western | dressed | spot | CE | 0.181983 |
|  | 0.886792 | 0 | primary | western | dressed | spot | CE | 0.223656 |
|  | 0.886792 | 0 | primary | western | intra | spot | CE | 0.287682 |
|  | 0.886792 | 0 | primary | western | dressed | spot | CE | 0.331647 |
| Szucs et al. (2014) | 0.921569 | 0 | primary | western | intra | digit | UWM | 0.331647 |
|  | 0.921569 | 0 | primary | western | intra | others | UWM | 0.266108 |
|  | 0.921569 | 0 | primary | western | intra | others | UWM | 0.140926 |
|  | 0.921569 | 0 | primary | western | intra | spot | VS | 0.522984 |
|  | 0.921569 | 0 | primary | western | intra | others | VS | 0.376886 |
| Maennamaa et al. (2012) | 0.943548 | 0 | primary | western | intra | block | VS | 0.354093 |
|  | 0.943548 | 0 | primary | western | intra | others | PL | 0.15114 |
|  | 0.943548 | 0 | primary | western | intra | others | PL | 0.244774 |
|  | 0.943548 | 0 | primary | western | intra | others | PL | 0.100335 |
|  | 0.943548 | 0 | primary | western | intra | others | PL | 0.213171 |
|  | 0.943548 | 0 | primary | western | intra | block | VS | 0.51007 |
|  | 0.943548 | 0 | primary | western | intra | others | PL | 0.100335 |
|  | 0.943548 | 0 | primary | western | intra | others | PL | 0.244774 |
|  | 0.943548 | 0 | primary | western | intra | others | PL | 0.060072 |
|  | 0.943548 | 0 | primary | western | intra | others | PL | 0.40006 |
|  | 0.943548 | 0 | primary | western | dressed | block | VS | 0.647523 |
|  | 0.943548 | 0 | primary | western | dressed | others | PL | 0.181983 |
|  | 0.943548 | 0 | primary | western | dressed | others | PL | 0.287682 |
|  | 0.943548 | 0 | primary | western | dressed | others | PL | 0.090244 |
|  | 0.943548 | 0 | primary | western | dressed | others | PL | 0.40006 |
|  | 0.943548 | 0 | primary | western | intra | block | VS | 0.287682 |
|  | 0.943548 | 0 | primary | western | intra | others | PL | 0.070115 |
|  | 0.943548 | 0 | primary | western | intra | others | PL | 0.266108 |
|  | 0.943548 | 0 | primary | western | intra | others | PL | 0 |
|  | 0.943548 | 0 | primary | western | intra | others | PL | 0.223656 |
| Jenks et al. (2012) |  | 1 | primary | western | dressed | digit | PL | 0.287682 |
|  |  | 1 | primary | western | dressed | block | VS | 0.725005 |
|  |  | 1 | primary | western | dressed | digit | CE | 0.708921 |
| De smedt et al. (2008) | 0.785714 | 1 | primary | western | intra | others | PL | 0.4118 |
|  | 0.785714 | 1 | primary | western | intra | block | VS | 0.120581 |
|  | 0.785714 | 1 | primary | western | intra | others | CE | -0.110447 |
|  | 0.785714 | 1 | primary | western | intra | others | CE | 0.331647 |
|  | 0.785714 | 1 | primary | western | intra | others | PL | 0.266108 |
|  | 0.785714 | 1 | primary | western | intra | block | VS | 0.202733 |
|  | 0.785714 | 1 | primary | western | intra | others | CE | 0.110447 |
|  | 0.785714 | 1 | primary | western | intra | others | CE | 0.15114 |
|  | 0.785714 | 1 | primary | western | intra | others | PL | 0.647523 |
|  | 0.785714 | 1 | primary | western | intra | block | VS | 0.459897 |
|  | 0.785714 | 1 | primary | western | intra | others | CE | 0.070115 |
|  | 0.785714 | 1 | primary | western | intra | others | CE | 0.677666 |
|  | 0.785714 | 1 | primary | western | dressed | others | PL | 0.497311 |
|  | 0.785714 | 1 | primary | western | dressed | block | VS | 0.459897 |
|  | 0.785714 | 1 | primary | western | dressed | others | CE | -0.192337 |
|  | 0.785714 | 1 | primary | western | dressed | others | CE | 0.181983 |
|  | 0.785714 | 0 | primary | western | intra | others | PL | 0.590145 |
|  | 0.785714 | 0 | primary | western | intra | block | VS | 0.213171 |
|  | 0.785714 | 0 | primary | western | intra | others | CE | 0.342828 |
|  | 0.785714 | 0 | primary | western | intra | others | CE | 0.320545 |
|  | 0.785714 | 0 | primary | western | intra | others | PL | 0.4847 |
|  | 0.785714 | 0 | primary | western | intra | block | VS | -0.13074 |
|  | 0.785714 | 0 | primary | western | intra | others | CE | 0.4847 |
|  | 0.785714 | 0 | primary | western | intra | others | CE | 0.213171 |
|  | 0.785714 | 0 | primary | western | intra | others | PL | 0.677666 |
|  | 0.785714 | 0 | primary | western | intra | block | VS | 0.244774 |
|  | 0.785714 | 0 | primary | western | intra | others | CE | 0.365444 |
|  | 0.785714 | 0 | primary | western | intra | others | CE | 0.171667 |
|  | 0.785714 | 0 | primary | western | dressed | others | PL | 0.693147 |
|  | 0.785714 | 0 | primary | western | dressed | block | VS | 0.223656 |
|  | 0.785714 | 0 | primary | western | dressed | others | CE | 0.435611 |
|  | 0.785714 | 0 | primary | western | dressed | others | CE | 0.171667 |
| Liu (2019) | 1.133333 | 1 | primary | eastern | dressed | others | UWM | 0.3977 |
| Traff et al.(2018) | 1.11811 | 0 | primary | western | intra | others | PL | 0.080171 |
|  | 1.11811 | 0 | primary | western | intra | others | PL | -0.192337 |
|  | 1.11811 | 0 | primary | western | intra | others | PL | -0.161387 |
|  | 1.11811 | 0 | primary | western | intra | others | PL | 0.192337 |
|  | 1.11811 | 0 | primary | western | intra | others | PL | 0.234189 |
|  | 1.11811 | 0 | primary | western | intra | others | PL | 0.161387 |
|  | 1.11811 | 0 | primary | western | dressed | others | PL | 0.255413 |
| Geary (2011) | 0.851852 | 0 | primary | western | intra | others | CE | 0.223656 |
|  | 0.851852 | 0 | primary | western | intra | others | CE | 0.276864 |
|  | 0.851852 | 0 | primary | western | intra | others | CE | 0.388423 |
|  | 0.851852 | 0 | primary | western | intra | others | CE | 0.110447 |
|  | 0.851852 | 0 | primary | western | intra | others | CE | 0.459897 |
|  | 0.851852 | 0 | primary | western | intra | others | CE | 0.459897 |
|  | 0.851852 | 0 | primary | western | intra | others | PL | 0.140926 |
|  | 0.851852 | 0 | primary | western | intra | others | PL | 0.13074 |
|  | 0.851852 | 0 | primary | western | intra | others | PL | 0.354093 |
|  | 0.851852 | 0 | primary | western | intra | others | PL | 0 |
|  | 0.851852 | 0 | primary | western | intra | others | PL | 0.459897 |
|  | 0.851852 | 0 | primary | western | intra | others | PL | 0.320545 |
|  | 0.851852 | 0 | primary | western | intra | block | VS | 0.120581 |
|  | 0.851852 | 0 | primary | western | intra | block | VS | 0.266108 |
|  | 0.851852 | 0 | primary | western | intra | block | VS | 0.213171 |
|  | 0.851852 | 0 | primary | western | intra | block | VS | 0.223656 |
|  | 0.851852 | 0 | primary | western | intra | block | VS | 0.234189 |
|  | 0.851852 | 0 | primary | western | intra | block | VS | 0.40006 |
| Greiner de Magalhães (2021) | 1.933333 | 0 | primary | western | intra | sentence | VS | 0.472231 |
|  | 1.933333 | 0 | primary | western | intra | sentence | VS | 0.549306 |
| Swanson (2006) | 1.22807 | 0 | primary | western | intra | digit | PL | 0.13074 |
|  | 1.22807 | 0 | primary | western | intra | others | PL | 0.15114 |
|  | 1.22807 | 0 | primary | western | intra | others | PL | 0.213171 |
|  | 1.22807 | 0 | primary | western | intra | digit | CE | 0.255413 |
|  | 1.22807 | 0 | primary | western | intra | sentence | CE | 0.435611 |
|  | 1.22807 | 0 | primary | western | intra | others | CE | 0.40006 |
|  | 1.22807 | 0 | primary | western | intra | block | VS | 0.213171 |
|  | 1.22807 | 0 | primary | western | intra | others | VS | 0.365444 |
|  | 1.22807 | 0 | primary | western | intra | digit | PL | 0.090244 |
|  | 1.22807 | 0 | primary | western | intra | others | PL | 0.15114 |
|  | 1.22807 | 0 | primary | western | intra | others | PL | 0.202733 |
|  | 1.22807 | 0 | primary | western | intra | digit | CE | 0.234189 |
|  | 1.22807 | 0 | primary | western | intra | sentence | CE | 0.435611 |
|  | 1.22807 | 0 | primary | western | intra | others | CE | 0.388423 |
|  | 1.22807 | 0 | primary | western | intra | block | VS | 0.255413 |
|  | 1.22807 | 0 | primary | western | intra | others | VS | 0.354093 |
| Wong & Ho (2017) | 1.155172 | 0 | primary | eastern | intra | others | PL | 0.037017 |
|  | 1.155172 | 0 | primary | eastern | intra | others | PL | -0.171667 |
|  | 1.155172 | 0 | primary | eastern | intra | others | PL | 0.304034 |
|  | 1.155172 | 0 | primary | eastern | intra | others | PL | 0.260753 |
|  | 1.155172 | 0 | primary | eastern | intra | others | PL | 0.372298 |
|  | 1.155172 | 0 | primary | eastern | dressed | others | PL | 0.375737 |
|  | 1.155172 | 0 | primary | eastern | intra | others | PL | 0.21736 |
|  | 1.155172 | 0 | primary | eastern | intra | block | VS | -0.266108 |
|  | 1.155172 | 0 | primary | eastern | intra | block | VS | -0.228917 |
|  | 1.155172 | 0 | primary | eastern | intra | block | VS | 0.124641 |
|  | 1.155172 | 0 | primary | eastern | intra | block | VS | 0.206903 |
|  | 1.155172 | 0 | primary | eastern | intra | block | VS | 0.220506 |
|  | 1.155172 | 0 | primary | eastern | dressed | block | VS | 0.201691 |
|  | 1.155172 | 0 | primary | eastern | intra | block | VS | 0.126673 |
|  | 1.155172 | 0 | primary | eastern | intra | digit | CE | -0.095287 |
|  | 1.155172 | 0 | primary | eastern | intra | digit | CE | -0.216312 |
|  | 1.155172 | 0 | primary | eastern | intra | digit | CE | 0.244774 |
|  | 1.155172 | 0 | primary | eastern | intra | digit | CE | 0.234189 |
|  | 1.155172 | 0 | primary | eastern | intra | digit | CE | 0.206903 |
|  | 1.155172 | 0 | primary | eastern | dressed | digit | CE | 0.270403 |
|  | 1.155172 | 0 | primary | eastern | intra | digit | CE | 0.238417 |
| Mabbott & Bisanz (2008) |  | 1 | primary | western | intra | digit | UWM | 0.244774 |
|  |  | 1 | primary | western | intra | digit | UWM | 0.13074 |
|  |  | 1 | primary | western | intra | digit | UWM | 0.020003 |
|  |  | 1 | primary | western | intra | digit | UWM | 0.120581 |
|  |  | 1 | primary | western | intra | digit | UWM | 0.13074 |
|  |  | 1 | primary | western | intra | operation | UWM | 0.435611 |
|  |  | 1 | primary | western | intra | operation | UWM | 0.435611 |
|  |  | 1 | primary | western | intra | operation | UWM | 0.234189 |
|  |  | 1 | primary | western | intra | operation | UWM | 0.213171 |
|  |  | 1 | primary | western | intra | operation | UWM | 0.497311 |
| Fuchs et al. (2012) | 0.912046 | 0 | primary | western | intra | sentence | UWM | 0.213171 |
|  | 0.912046 | 0 | primary | western | dressed | sentence | UWM | 0.30952 |
|  | 0.912046 | 0 | primary | western | intra | sentence | UWM | 0.213171 |
|  | 0.912046 | 0 | primary | western | dressed | sentence | UWM | 0.40006 |
|  | 0.912046 | 0 | primary | western | intra | sentence | UWM | 0.266108 |
|  | 0.912046 | 0 | primary | western | intra | digit | UWM | 0.120581 |
|  | 0.912046 | 0 | primary | western | dressed | digit | UWM | 0.234189 |
|  | 0.912046 | 0 | primary | western | intra | digit | UWM | 0.266108 |
|  | 0.912046 | 0 | primary | western | dressed | digit | UWM | 0.298566 |
|  | 0.912046 | 0 | primary | western | intra | digit | UWM | 0.276864 |
| Swanson (2006) | 0.927711 | 0 | primary | western | intra | digit | PL | 0.060072 |
|  | 0.927711 | 0 | primary | western | intra | block | VS | 0.15114 |
|  | 0.927711 | 0 | primary | western | intra | others | CE | 0.070115 |
|  | 0.927711 | 0 | primary | western | dressed | digit | PL | 0.15114 |
|  | 0.927711 | 0 | primary | western | dressed | block | VS | -0.01 |
|  | 0.927711 | 0 | primary | western | dressed | others | CE | 0.234189 |
| Reeve et al. (2018) |  | 0 | primary | western | intra | block | VS | 0.51007 |
|  |  | 0 | primary | western | intra | block | VS | 0.423649 |
| Li & Geary (2013) |  | 0 | primary | western | intra | others | CE | 0.549306 |
|  |  | 0 | primary | western | intra | others | PL | 0.276864 |
|  |  | 0 | primary | western | intra | others | VS | 0.40006 |
|  |  | 0 | primary | western | intra | block | VS | 0.30952 |
| Jordan et al. (2013) | 0.897533 | 0 | primary | western | intra | spot | UWM | 0.358622 |
|  | 0.897533 | 0 | primary | western | intra | spot | UWM | 0.129723 |
|  | 0.897533 | 0 | primary | western | intra | spot | UWM | 0.232079 |
|  | 0.897533 | 0 | primary | western | intra | spot | UWM | 0.363166 |
|  | 0.897533 | 0 | primary | western | intra | spot | UWM | 0.34845 |
|  | 0.897533 | 0 | primary | western | intra | spot | UWM | 0.370009 |
|  | 0.897533 | 0 | primary | western | intra | spot | UWM | 0.355224 |
| Lee & Peh (2008) | 2.138889 | 0 | primary | eastern | dressed | others | PL | 0.206068 |
|  | 2.138889 | 0 | primary | eastern | dressed | block | VS | 0.262465 |
|  | 2.138889 | 0 | primary | eastern | dressed | sentence | CE | 0.197218 |
| Meyer et al. (2010) | 1.823529 | 0 | primary | western | intra | spot | CE | 0.192337 |
|  | 1.823529 | 0 | primary | western | intra | digit | CE | 0.090244 |
|  | 1.823529 | 0 | primary | western | intra | digit | PL | 0.100335 |
|  | 1.823529 | 0 | primary | western | intra | block | VS | 0.060072 |
|  | 1.823529 | 0 | primary | western | both | spot | CE | 0.53606 |
|  | 1.823529 | 0 | primary | western | both | digit | CE | 0.110447 |
|  | 1.823529 | 0 | primary | western | both | digit | PL | 0.435611 |
|  | 1.823529 | 0 | primary | western | both | block | VS | 0.13074 |
|  | 1.083333 | 0 | primary | western | intra | spot | CE | 0.213171 |
|  | 1.083333 | 0 | primary | western | intra | digit | CE | 0.298566 |
|  | 1.083333 | 0 | primary | western | intra | digit | PL | 0.161387 |
|  | 1.083333 | 0 | primary | western | intra | block | VS | 0.472231 |
|  | 1.083333 | 0 | primary | western | both | spot | CE | 0.181983 |
|  | 1.083333 | 0 | primary | western | both | digit | CE | 0.161387 |
|  | 1.083333 | 0 | primary | western | both | digit | PL | 0.192337 |
|  | 1.083333 | 0 | primary | western | both | block | VS | 0.40006 |
| Wu et al. (2017) | 0.918605 | 0 | primary | western | both | digit | PL | 0.331647 |
|  | 0.918605 | 0 | primary | western | intra | digit | PL | 0.15114 |
|  | 0.918605 | 0 | primary | western | both | block | VS | 0.376886 |
|  | 0.918605 | 0 | primary | western | intra | block | VS | 0.376886 |
|  | 0.918605 | 0 | primary | western | both | spot | CE | 0.447692 |
|  | 0.918605 | 0 | primary | western | intra | spot | CE | 0.472231 |
|  | 0.918605 | 0 | primary | western | both | digit | CE | 0.255413 |
|  | 0.918605 | 0 | primary | western | intra | digit | CE | 0.30952 |
| Yip et al. (2020) | 1.594937 | 1 | primary | eastern |  |  | VS | 0.435611 |
|  | 1.594937 | 1 | primary | eastern |  |  | VS | 0.447692 |
|  | 1.594937 | 1 | primary | eastern | intra |  | VS | 0.423649 |
|  | 1.594937 | 1 | primary | eastern | dressed |  | VS | 0.181983 |
|  | 1.594937 | 1 | primary | eastern |  | block | UWM | 0.522984 |
|  | 1.594937 | 1 | primary | eastern |  | block | UWM | 0.522984 |
|  | 1.594937 | 1 | primary | eastern | intra | block | UWM | 0.590145 |
|  | 1.594937 | 1 | primary | eastern | dressed | block | UWM | 0.276864 |
| Fuchs et al. (2010) | 1.173913 | 0 | primary |  | intra | sentence | CE | 0.110447 |
|  | 1.173913 | 0 | primary |  | intra | digit | CE | 0.202733 |
|  | 1.173913 | 0 | primary |  | intra | others | PL | -0.090244 |
|  | 1.173913 | 0 | primary |  | intra | block | VS | 0.050042 |
|  | 1.173913 | 0 | primary |  | dressed | sentence | CE | 0.192337 |
|  | 1.173913 | 0 | primary |  | dressed | digit | CE | -0.050042 |
|  | 1.173913 | 0 | primary |  | dressed | others | PL | -0.030009 |
|  | 1.173913 | 0 | primary |  | dressed | block | VS | 0.060072 |
| Fuchs et al. (2020) | 0.960784 | 0 | primary | western | intra | sentence | CE | 0.223656 |
| Keeler & Swanson (2001) | 2 | 1 | primary | western |  | digit | PL | 0.376886 |
|  | 2 | 1 | primary | western |  | digit | PL | 0.4847 |
|  | 2 | 1 | primary | western |  | digit | PL | 0.459897 |
|  | 2 | 1 | primary | western |  | digit | PL | 0.213171 |
|  | 2 | 1 | primary | western |  | others | VS | 0.423649 |
|  | 2 | 1 | primary | western |  | others | VS | 0.342828 |
|  | 2 | 1 | primary | western |  | others | VS | 0.213171 |
|  | 2 | 1 | primary | western |  | digit | PL | 0.741416 |
|  | 2 | 1 | primary | western |  | others | VS | 0.604156 |
| Siegler et al.(2012) |  | 0 | high | western | intra | digit | UWM | 0.354093 |
|  |  | 0 | high | western | dressed | digit | UWM | 0.40006 |
| Lukowski et al. (2014) | 0.675042 | 0 |  | western | dressed | block | VS | 0.342828 |
|  | 0.675042 | 0 |  | western | intra | block | VS | 0.320545 |
|  | 0.675042 | 0 |  | western | intra | block | VS | 0.298566 |
|  | 0.675042 | 0 |  | western | dressed | digit | PL | 0.331647 |
|  | 0.675042 | 0 |  | western | intra | digit | PL | 0.266108 |
|  | 0.675042 | 0 |  | western | intra | digit | PL | 0.181983 |
| St Clair-Thompson & Gathercole (2006) | 1.888889 | 0 | primary | western |  | digit | CE | 0.447692 |
|  | 1.888889 | 0 | primary | western |  | others | CE | 0.255413 |
|  | 1.888889 | 0 | primary | western |  | others | CE | 0.354093 |
|  | 1.888889 | 0 | primary | western |  | others | CE | 0.604156 |
|  | 1.888889 | 0 | primary | western |  | others | CE | 0.15114 |
|  | 1.888889 | 0 | primary | western |  | block | CE | 0.320545 |
|  | 1.888889 | 0 | primary | western |  | sentence | UWM | 0.255413 |
|  | 1.888889 | 0 | primary | western |  | digit | UWM | 0.090244 |
|  | 1.888889 | 0 | primary | western |  | others | UWM | 0.522984 |
|  | 1.888889 | 0 | primary | western |  | others | UWM | 0.472231 |
| Cormier et al. (2017) | 0.964637 | 0 |  | western | intra | others | UWM | 0.192337 |
|  | 0.964637 | 0 |  | western | dressed | others | UWM | 0.255413 |
|  | 0.988072 | 0 |  | western | intra | others | UWM | 0.171667 |
|  | 0.988072 | 0 |  | western | dressed | others | UWM | 0.181983 |
|  | 1.004008 | 0 |  | western | intra | others | UWM | 0.120581 |
|  | 1.004008 | 0 |  | western | dressed | others | UWM | 0.13074 |
|  | 0.960784 | 0 |  | western | intra | others | UWM | 0.202733 |
|  | 0.960784 | 0 |  | western | dressed | others | UWM | 0.161387 |
|  | 1 | 0 |  | western | intra | others | UWM | 0.120581 |
|  | 1 | 0 |  | western | dressed | others | UWM | 0.161387 |
|  | 1.020202 | 0 |  | western | intra | others | UWM | 0.181983 |
|  | 1.020202 | 0 |  | western | dressed | others | UWM | 0.060072 |
|  | 1.008032 | 0 |  | western | intra | others | UWM | 0.120581 |
|  | 1.008032 | 0 |  | western | dressed | others | UWM | 0.161387 |
|  | 0.883239 | 0 |  | western | intra | others | UWM | 0.223656 |
|  | 0.883239 | 0 |  | western | dressed | others | UWM | 0.181983 |
|  | 0.992032 | 0 |  | western | intra | others | UWM | 0.244774 |
|  | 0.992032 | 0 |  | western | dressed | others | UWM | 0.140926 |
|  | 1.083333 | 0 |  | western | intra | others | UWM | -0.020003 |
|  | 1.083333 | 0 |  | western | dressed | others | UWM | 0.13074 |
|  | 1 | 0 |  | western | intra | others | UWM | 0.120581 |
|  | 1 | 0 |  | western | dressed | others | UWM | 0.202733 |
|  | 0.869159 | 0 |  | western | intra | others | UWM | 0.15114 |
|  | 0.869159 | 0 |  | western | dressed | others | UWM | 0.161387 |
|  | 0.876173 | 0 |  | western | intra | others | UWM | 0.192337 |
|  | 0.876173 | 0 |  | western | dressed | others | UWM | 0.15114 |
|  | 0.904762 | 0 |  | western | intra | others | UWM | 0.161387 |
|  | 0.904762 | 0 |  | western | dressed | others | UWM | 0.171667 |
| Metcalfe et al. (2013) | 0.85 | 0 | primary | western | intra | spot | CE | 0.266108 |
|  | 0.85 | 0 | primary | western | intra | digit | PL | 0.080171 |
|  | 0.85 | 0 | primary | western | intra | block | VS | 0.472231 |
|  | 0.85 | 0 | primary | western | dressed | spot | CE | 0.388423 |
|  | 0.85 | 0 | primary | western | dressed | digit | PL | 0.266108 |
|  | 0.85 | 0 | primary | western | dressed | block | VS | 0.255413 |
| Swanson et al. (2013) | 1.022222 | 1 | primary | western | dressed | others | UWM | 0.459897 |
|  | 1.022222 | 1 | primary | western | dressed | others | UWM | 0.298566 |
| Swanson et al. (2008) | 1.097222 | 1 | primary | western | dressed | others | VS | 0.376886 |
|  | 1.097222 | 1 | primary | western | dressed | others | PL | 0.435611 |
|  | 1.097222 | 1 | primary | western | dressed | sentence | CE | 0.590145 |
|  | 1.097222 | 1 | primary | western | intra | others | VS | 0.354093 |
|  | 1.097222 | 1 | primary | western | intra | others | PL | 0.4847 |
|  | 1.097222 | 1 | primary | western | intra | sentence | CE | 0.590145 |
|  | 1.097222 | 1 | primary | western | dressed | others | VS | 0.342828 |
|  | 1.097222 | 1 | primary | western | dressed | others | PL | 0.472231 |
|  | 1.097222 | 1 | primary | western | dressed | sentence | CE | 0.604156 |
| Matejko et al. (2017) | 1.315789 | 0 | primary | western | intra | spot | VS | 0.020003 |
|  | 1.315789 | 0 | primary | western | intra | spot | VS | 0.030009 |
|  | 1.315789 | 0 | primary | western | intra | spot | VS | 0.244774 |
|  | 1.315789 | 0 | primary | western | intra | spot | VS | -0.060072 |
|  | 1.315789 | 0 | primary | western | intra | spot | VS | -0.522984 |
|  | 1.315789 | 0 | primary | western | intra | spot | VS | -0.342828 |
| De Smedt et al. (2010) |  | 0 | primary | western | intra | others | PL | 0.020003 |
|  |  | 0 | primary | western | intra | others | PL | 0.13074 |
|  |  | 0 | primary | western | intra | others | PL | -0.223656 |
| Viterbori et al. (2015) | 1.303571 | 0 | primary | western | intra | others | CE | 0.15114 |
|  | 1.303571 | 0 | primary | western | intra | others | CE | 0.181983 |
|  | 1.303571 | 0 | primary | western | intra | others | CE | 0.140926 |
|  | 1.303571 | 0 | primary | western | intra | others | CE | 0.331647 |
|  | 1.303571 | 0 | primary | western | intra | others | CE | 0.070115 |
|  | 1.303571 | 0 | primary | western | intra | others | CE | 0.331647 |
|  | 1.303571 | 0 | primary | western | intra | others | CE | 0.161387 |
|  | 1.303571 | 0 | primary | western | intra | others | CE | 0.223656 |
|  | 1.303571 | 0 | primary | western | intra | others | CE | 0.181983 |
|  | 1.303571 | 0 | primary | western | intra | others | CE | 0.287682 |
|  | 1.303571 | 0 | primary | western | intra | others | CE | 0.080171 |
|  | 1.303571 | 0 | primary | western | intra | others | CE | 0.202733 |
|  | 1.303571 | 0 | primary | western | dressed | others | CE | 0.223656 |
|  | 1.303571 | 0 | primary | western | dressed | others | CE | 0.342828 |
|  | 1.303571 | 0 | primary | western | dressed | others | CE | 0.213171 |
|  | 1.303571 | 0 | primary | western | dressed | others | CE | 0.4118 |
|  | 1.303571 | 0 | primary | western | dressed | others | CE | 0.181983 |
|  | 1.303571 | 0 | primary | western | dressed | others | CE | 0.192337 |
|  | 1.408163 | 0 | primary | western | intra | others | CE | 0.100335 |
|  | 1.408163 | 0 | primary | western | intra | others | CE | 0.120581 |
|  | 1.408163 | 0 | primary | western | intra | others | CE | 0.181983 |
|  | 1.408163 | 0 | primary | western | intra | others | CE | 0.234189 |
|  | 1.408163 | 0 | primary | western | intra | others | CE | 0.120581 |
|  | 1.408163 | 0 | primary | western | intra | others | CE | 0.192337 |
|  | 1.408163 | 0 | primary | western | intra | others | CE | 0.030009 |
|  | 1.408163 | 0 | primary | western | intra | others | CE | 0.080171 |
|  | 1.408163 | 0 | primary | western | intra | others | CE | 0.276864 |
|  | 1.408163 | 0 | primary | western | intra | others | CE | 0.376886 |
|  | 1.408163 | 0 | primary | western | intra | others | CE | 0.223656 |
|  | 1.408163 | 0 | primary | western | intra | others | CE | 0.276864 |
|  | 1.408163 | 0 | primary | western | dressed | others | CE | 0.01 |
|  | 1.408163 | 0 | primary | western | dressed | others | CE | 0.171667 |
|  | 1.408163 | 0 | primary | western | dressed | others | CE | 0.354093 |
|  | 1.408163 | 0 | primary | western | dressed | others | CE | 0.365444 |
|  | 1.408163 | 0 | primary | western | dressed | others | CE | 0.30952 |
|  | 1.408163 | 0 | primary | western | dressed | others | CE | 0.287682 |
| Cornoldi (2015) | 0.927536 | 0 | primary | western | dressed | others | UWM | 0.298566 |
| Swanson et al. (2019) | 0.950495 | 0 | primary | western | dressed | others | CE | 0.161387 |
|  | 0.950495 | 0 | primary | western | dressed | others | CE | 0.13074 |
|  | 0.950495 | 0 | primary | western | dressed | others | CE | 0.342828 |
| Song et al. (2011) | 1.418605 | 0 | primary | eastern | dressed | operation | PL | 0.0408 |
|  | 1.418605 | 0 | primary | eastern | dressed | operation | PL | -0.073 |
|  | 1.418605 | 0 | primary | eastern | dressed | operation | PL | 0.2042 |
|  | 1.418605 | 0 | primary | eastern | dressed | others | VS | 0.2757 |
|  | 1.418605 | 0 | primary | eastern | dressed | others | VS | 0.2113 |
|  | 1.418605 | 0 | primary | eastern | dressed | others | VS | 0.3157 |
| Khng & LEE (2009) | 0.869048 | 0 | middle | eastern | dressed | others | UWM | 0.320545 |
| Navarro et al. (2011) |  | 0 |  | western | intra | digit | CE | 0.637215 |
|  |  | 0 |  | western | intra | digit | CE | 0.243713 |
|  |  | 0 |  | western | intra | others | PL | 0.450123 |
|  |  | 0 |  | western | intra | others | PL | 0.252215 |
|  |  | 0 |  | western | intra | others | PL | 0.452559 |
| Ng et al. (2017) | 0.942029 | 0 | primary | eastern | intra | sentence | UWM | 0.140926 |
|  | 0.942029 | 0 | primary | eastern | intra | sentence | UWM | 0.070115 |
| Fuchs et al. (2015) | 0.925234 | 0 | primary | western | intra | spot | UWM | 0.354093 |
|  | 0.925234 | 0 | primary | western | intra | sentence | UWM | 0.365444 |
|  | 0.925234 | 0 | primary | western | dressed | spot | UWM | 0.459897 |
|  | 0.925234 | 0 | primary | western | dressed | sentence | UWM | 0.53606 |
| Zhang et al. (2017) | 1.076923 | 0 | primary | western | intra | digit | UWM | 0.161387 |
|  | 1.076923 | 0 | primary | western | intra | digit | UWM | 0.234189 |
|  | 1.076923 | 0 | primary | western | intra | digit | UWM | 0.15114 |
|  | 1.076923 | 0 | primary | western | dressed | digit | UWM | 0.15114 |
|  | 1.076923 | 0 | primary | western | intra | digit | UWM | 0.202733 |
|  | 1.076923 | 0 | primary | western | intra | digit | UWM | 0.202733 |
|  | 1.076923 | 0 | primary | western | intra | digit | UWM | 0.298566 |
|  | 1.076923 | 0 | primary | western | intra | digit | UWM | 0.223656 |
|  | 1.076923 | 0 | primary | western | dressed | digit | UWM | 0.266108 |
|  | 1.076923 | 0 | primary | western | intra | digit | UWM | 0.287682 |
| Spencer et al. (2020) | 0.60885 | 0 | primary | western | dressed | sentence | UWM | 0.414161 |
|  | 0.60885 | 0 | primary | western | intra | sentence | UWM | 0.189227 |
|  | 0.60885 | 0 | primary | western | intra | sentence | UWM | 0.148073 |
|  | 0.60885 | 0 | primary | western | intra | sentence | UWM | 0.138886 |
|  | 0.60885 | 0 | primary | western | intra | sentence | UWM | 0.148073 |
|  | 0.60885 | 0 | primary | western | intra | sentence | UWM | 0.140926 |
|  | 0.60885 | 0 | primary | western | intra | sentence | UWM | 0.052047 |
|  | 0.60885 | 0 | primary | western | dressed | sentence | UWM | 0.41298 |
|  | 0.60885 | 0 | primary | western | dressed | sentence | UWM | 0.265036 |
|  | 0.60885 | 0 | primary | western | dressed | spot | UWM | 0.289854 |
|  | 0.60885 | 0 | primary | western | intra | spot | UWM | 0.277943 |
|  | 0.60885 | 0 | primary | western | intra | spot | UWM | 0.219457 |
|  | 0.60885 | 0 | primary | western | intra | spot | UWM | 0.281183 |
|  | 0.60885 | 0 | primary | western | intra | spot | UWM | 0.21736 |
|  | 0.60885 | 0 | primary | western | intra | spot | UWM | 0.227863 |
|  | 0.60885 | 0 | primary | western | intra | spot | UWM | 0.233134 |
|  | 0.60885 | 0 | primary | western | dressed | spot | UWM | 0.342828 |
|  | 0.60885 | 0 | primary | western | dressed | spot | UWM | 0.277943 |
| Barnes et al. (2014) | 0.710526 | 1 | primary | western | intra | block | VS | 0.632833 |
|  | 0.710526 | 1 | primary | western | intra | block | VS | 0.287682 |
|  | 0.710526 | 1 | primary | western | intra | block | VS | 0.331647 |
|  | 0.710526 | 1 | primary | western | intra | others | PL | 0.590145 |
|  | 0.710526 | 1 | primary | western | intra | others | PL | 0.497311 |
|  | 0.710526 | 1 | primary | western | intra | others | PL | 0.342828 |
| Jose et al. (2017) | 0.94186 | 0 | primary | western | intra | others | UWM | 0.40006 |
|  | 0.94186 | 0 | primary | western | intra | others | UWM | 0.423649 |
|  | 0.94186 | 0 | primary | western | intra | others | UWM | 0.342828 |
| Jansen et al. (2013) | 1.148148 | 0 | middle | western | intra | sentence | PL | 0.012001 |
|  | 1.148148 | 0 | middle | western | intra | sentence | PL | 0.006 |
|  | 1.148148 | 0 | middle | western | intra | sentence | PL | 0.134811 |
|  | 1.148148 | 0 | middle | western | intra | sentence | PL | 0.164467 |
|  | 1.148148 | 0 | middle | western | intra | others | PL | 0.294204 |
|  | 1.148148 | 0 | middle | western | intra | others | PL | 0.292028 |
|  | 1.148148 | 0 | middle | western | intra | others | PL | 0.160361 |
|  | 1.148148 | 0 | middle | western | intra | others | PL | 0.254346 |
|  | 1.148148 | 0 | middle | western | intra | digit | PL | 0.20586 |
|  | 1.148148 | 0 | middle | western | intra | digit | PL | 0.087221 |
|  | 1.148148 | 0 | middle | western | intra | digit | PL | 0.028007 |
|  | 1.148148 | 0 | middle | western | intra | digit | PL | 0.005 |
|  | 1.148148 | 0 | middle | western | intra | block | VS | 0.474714 |
|  | 1.148148 | 0 | middle | western | intra | block | VS | 0.301845 |
|  | 1.148148 | 0 | middle | western | intra | block | VS | 0.023004 |
|  | 1.148148 | 0 | middle | western | intra | block | VS | -0.006 |
| Andersson (2008) | 0.915789 | 1 | primary | western | intra | block | PL | 0.120581 |
|  | 0.915789 | 1 | primary | western | intra | block | PL | 0.266108 |
|  | 0.915789 | 1 | primary | western | intra | block | PL | 0.060072 |
|  | 0.915789 | 1 | primary | western | intra | block | PL | 0.171667 |
|  | 0.915789 | 1 | primary | western | intra | block | PL | 0.120581 |
|  | 0.915789 | 1 | primary | western | dressed | block | PL | 0.223656 |
|  | 0.915789 | 1 | primary | western | dressed | block | PL | 0.244774 |
|  | 0.915789 | 1 | primary | western | dressed | block | PL | 0.244774 |
| Swanson & Sachse-Lee (2001) |  | 1 | primary | western | dressed | others | VS | 0.234189 |
|  |  | 1 | primary | western | dressed | others | VS | 0.181983 |
|  |  | 1 | primary | western | dressed | others | VS | -0.01 |
|  |  | 1 | primary | western | dressed | others | VS | 0.13074 |
|  |  | 1 | primary | western | dressed | others | VS | 0.100335 |
|  |  | 1 | primary | western | dressed | others | VS | -0.266108 |
|  |  | 1 | primary | western | dressed | others | VS | 0.202733 |
|  |  | 1 | primary | western | dressed | others | PL | 0.522984 |
|  |  | 1 | primary | western | dressed | others | PL | 0.100335 |
|  |  | 1 | primary | western | dressed | others | PL | -0.020003 |
|  |  | 1 | primary | western | dressed | others | PL | 0.255413 |
|  |  | 1 | primary | western | dressed | others | PL | 0.4118 |
|  |  | 1 | primary | western | dressed | others | PL | 0.51007 |
|  |  | 1 | primary | western | dressed | others | PL | -0.030009 |
|  |  | 1 | primary | western | dressed | others | PL | 0.331647 |
|  |  | 1 | primary | western | dressed | others | PL | 0.202733 |
|  |  | 1 | primary | western | dressed | others | PL | 0.100335 |
|  |  | 1 | primary | western | dressed | others | PL | 0.472231 |
|  |  | 1 | primary | western | dressed | others | PL | 0.497311 |
|  |  | 1 | primary | western | dressed | others | PL | 0.497311 |
|  |  | 1 | primary | western | dressed | others | PL | 0.171667 |
| Passolunghi (2015) | 1.625 | 1 | primary | western | intra | sentence | PL | 0.161387 |
|  | 1.625 | 1 | primary | western | intra | digit | UWM | 0.060072 |
|  | 1.625 | 1 | primary | western | intra | block | VS | 0.181983 |
|  | 1.625 | 1 | primary | western | intra | others | PL | 0.40006 |
|  | 1.625 | 1 | primary | western | intra | others | CE | 0.320545 |
|  | 1.625 | 1 | primary | western | intra | sentence | PL | 0.181983 |
|  | 1.625 | 1 | primary | western | intra | digit | UWM | 0.030009 |
|  | 1.625 | 1 | primary | western | intra | block | VS | 0.331647 |
|  | 1.625 | 1 | primary | western | intra | others | PL | 0.223656 |
|  | 1.625 | 1 | primary | western | intra | others | CE | 0.30952 |
|  | 1.625 | 1 | primary | western | intra | sentence | PL | 0.342828 |
|  | 1.625 | 1 | primary | western | intra | digit | UWM | 0.213171 |
|  | 1.625 | 1 | primary | western | intra | block | VS | 0.266108 |
|  | 1.625 | 1 | primary | western | intra | others | PL | 0.365444 |
|  | 1.625 | 1 | primary | western | intra | others | CE | 0.40006 |
|  | 1.625 | 1 | primary | western | dressed | sentence | PL | 0.331647 |
|  | 1.625 | 1 | primary | western | dressed | digit | UWM | 0.171667 |
|  | 1.625 | 1 | primary | western | dressed | block | VS | 0.365444 |
|  | 1.625 | 1 | primary | western | dressed | others | PL | 0.244774 |
|  | 1.625 | 1 | primary | western | dressed | others | CE | 0.388423 |
|  | 1.625 | 1 | primary | western | dressed | sentence | PL | 0.234189 |
|  | 1.625 | 1 | primary | western | dressed | digit | UWM | 0.161387 |
|  | 1.625 | 1 | primary | western | dressed | block | VS | 0.388423 |
|  | 1.625 | 1 | primary | western | dressed | others | PL | 0.376886 |
|  | 1.625 | 1 | primary | western | dressed | others | CE | 0.342828 |
|  | 1.625 | 1 | primary | western | intra | sentence | PL | 0.192337 |
|  | 1.625 | 1 | primary | western | intra | digit | UWM | -0.192337 |
|  | 1.625 | 1 | primary | western | intra | block | VS | 0.020003 |
|  | 1.625 | 1 | primary | western | intra | others | PL | 0.276864 |
|  | 1.625 | 1 | primary | western | intra | others | CE | 0.276864 |
|  | 1.625 | 1 | primary | western | intra | sentence | PL | 0.365444 |
|  | 1.625 | 1 | primary | western | intra | digit | UWM | 0.202733 |
|  | 1.625 | 1 | primary | western | intra | block | VS | 0.287682 |
|  | 1.625 | 1 | primary | western | intra | others | PL | 0.223656 |
|  | 1.625 | 1 | primary | western | intra | others | CE | 0.223656 |
| Ching (2017) | 0.863636 | 0 | primary | eastern | dressed | digit | UWM | 0.243713 |
|  | 0.863636 | 0 | primary | eastern | intra | digit | UWM | 0.21108 |
|  | 0.863636 | 0 | primary | eastern | intra | digit | UWM | 0.237359 |
| Zhu et al. (2017) | 1.333333 | 0 | primary | eastern | dressed | others | UWM | 0.423649 |
|  | 1.333333 | 0 | primary | eastern | intra | others | UWM | 0.234189 |
|  | 1.333333 | 0 | primary | eastern | intra | others | UWM | 0.320545 |
|  | 1.448276 | 0 | primary | eastern | dressed | others | UWM | 0.020003 |
|  | 1.448276 | 0 | primary | eastern | intra | others | UWM | 0.13074 |
|  | 1.448276 | 0 | primary | eastern | intra | others | UWM | 0.234189 |
| Ramirez et al. (2016) |  | 0 | primary |  | dressed | others | UWM | 0.481945 |
| Friedman et al. (2014) |  | 0 | primary | western |  | others | UWM | 0.15114 |
| Hale et al. (2003) |  | 0 | primary | western |  | others | UWM | 0.331647 |
|  |  | 0 | primary | western |  | others | UWM | 0.40006 |
| Fuchs et al. (2008) | 0.897533 | 0 | primary | western | intra | sentence | PL | 0.223656 |
|  | 0.897533 | 0 | primary | western | intra | sentence | PL | 0.244774 |
|  | 0.897533 | 0 | primary | western | intra | sentence | PL | 0.244774 |
|  | 0.897533 | 0 | primary | western | dressed | sentence | PL | 0.40006 |
|  | 0.897533 | 0 | primary | western | dressed | sentence | PL | 0.354093 |
|  | 0.897533 | 0 | primary | western | dressed | sentence | PL | 0.331647 |
|  | 0.897533 | 0 | primary | western | dressed | sentence | PL | 0.320545 |
|  | 0.897533 | 0 | primary | western | intra | digit | UWM | 0.234189 |
|  | 0.897533 | 0 | primary | western | intra | digit | UWM | 0.287682 |
|  | 0.897533 | 0 | primary | western | intra | digit | UWM | 0.244774 |
|  | 0.897533 | 0 | primary | western | dressed | digit | UWM | 0.331647 |
|  | 0.897533 | 0 | primary | western | dressed | digit | UWM | 0.342828 |
|  | 0.897533 | 0 | primary | western | dressed | digit | UWM | 0.266108 |
|  | 0.897533 | 0 | primary | western | dressed | digit | UWM | 0.255413 |
| Resing et al. (2017) | 0.962264 | 0 | primary | western | intra | sentence | PL | -0.064088 |
|  | 0.962264 | 0 | primary | western | intra | others | VS | 0.085206 |
| Paul et al. (2019) | 1.405405 | 0 | primary | western | intra | block | VS | 0.255413 |
|  | 1.405405 | 0 | primary | western | intra | block | VS | 0.298566 |
|  | 1.405405 | 0 | primary | western | intra | block | VS | 0.140926 |
|  | 1.405405 | 0 | primary | western | intra | block | VS | 0.223656 |
|  | 1.405405 | 0 | primary | western | intra | block | VS | 0.161387 |
|  | 1.405405 | 0 | primary | western | intra | block | VS | 0.13074 |
|  | 1.405405 | 0 | primary | western | intra | block | VS | 0.320545 |
|  | 1.405405 | 0 | primary | western | intra | block | VS | 0.060072 |
| Kleemans et al. (2018) | 1.197368 | 0 |  | western | intra | digit | UWM | 0.350704 |
|  | 1.197368 | 0 |  | western | intra | digit | UWM | 0.262894 |
|  | 1.197368 | 0 |  | western | intra | digit | UWM | 0.311719 |
| Andersson (2010) |  | 1 | primary | western | intra | others | CE | 0.050042 |
|  |  | 1 | primary | western | intra | spot | PL | 0.110447 |
|  |  | 1 | primary | western | intra | digit | UWM | 0.181983 |
|  |  | 1 | primary | western | intra | others | CE | -0.040021 |
|  |  | 1 | primary | western | intra | spot | PL | 0.255413 |
|  |  | 1 | primary | western | intra | digit | UWM | -0.01 |
|  |  | 1 | primary | western | intra | others | CE | -0.030009 |
|  |  | 1 | primary | western | intra | spot | PL | 0.202733 |
|  |  | 1 | primary | western | intra | digit | UWM | 0.050042 |
|  |  | 1 | primary | western | intra | others | CE | -0.171667 |
|  |  | 1 | primary | western | intra | spot | PL | 0.13074 |
|  |  | 1 | primary | western | intra | digit | UWM | -0.040021 |
|  |  | 1 | primary | western | dressed | others | CE | -0.060072 |
|  |  | 1 | primary | western | dressed | spot | PL | 0.276864 |
|  |  | 1 | primary | western | dressed | digit | UWM | -0.020003 |
|  |  | 1 | primary | western | dressed | others | CE | -0.070115 |
|  |  | 1 | primary | western | dressed | spot | PL | 0.266108 |
|  |  | 1 | primary | western | dressed | digit | UWM | 0.070115 |
|  |  | 1 | primary | western | intra | others | CE | -0.080171 |
|  |  | 1 | primary | western | intra | spot | PL | 0.171667 |
|  |  | 1 | primary | western | intra | digit | UWM | 0.050042 |
|  |  | 1 | primary | western | intra | others | CE | -0.070115 |
|  |  | 1 | primary | western | intra | spot | PL | 0.234189 |
|  |  | 1 | primary | western | intra | digit | UWM | 0.070115 |
| Hecht et al. (2003) | 0.810345 | 0 | primary | western | intra | spot | UWM | 0.234189 |
|  | 0.810345 | 0 | primary | western | intra | spot | UWM | 0.56273 |
|  | 0.810345 | 0 | primary | western | intra | spot | UWM | 0.266108 |
|  | 0.810345 | 0 | primary | western | intra | spot | UWM | 0.423649 |
|  | 0.810345 | 0 | primary | western | intra | spot | UWM | 0.161387 |
|  | 0.810345 | 0 | primary | western | dressed | spot | UWM | 0.497311 |
| Passolunghi & Mammarella (2010) | 1.857143 | 0 | primary |  | both | digit | PL | 0 |
|  | 1.857143 | 0 | primary |  | both | others | PL | 0.21 |
|  | 1.857143 | 0 | primary |  | both | others | PL | 0.2524 |
|  | 1.857143 | 0 | primary |  | both | digit | PL | 0.1658 |
|  | 1.857143 | 0 | primary |  | both | digit | CE | 0.3717 |
|  | 1.857143 | 0 | primary |  | both | others | CE | 0.179 |
|  | 1.857143 | 0 | primary |  | both | block | VS | 0.3057 |
|  | 1.857143 | 0 | primary |  | both | block | VS | 0.9105 |
|  | 1.857143 | 0 | primary |  | both | block | VS | 0.6652 |
|  | 1.857143 | 0 | primary |  | both | others | VS | 0.273 |
|  | 1.6 | 0 | primary |  | both | digit | PL | 0.0576 |
|  | 1.6 | 0 | primary |  | both | others | PL | 0.1739 |
|  | 1.6 | 0 | primary |  | both | others | PL | 0.1121 |
|  | 1.6 | 0 | primary |  | both | digit | PL | -0.0187 |
|  | 1.6 | 0 | primary |  | both | digit | CE | 0.093 |
|  | 1.6 | 0 | primary |  | both | others | CE | 0.3008 |
|  | 1.6 | 0 | primary |  | both | block | VS | 0.3372 |
|  | 1.6 | 0 | primary |  | both | block | VS | 0.5052 |
|  | 1.6 | 0 | primary |  | both | block | VS | 0.3937 |
|  | 1.6 | 0 | primary |  | both | block | VS | 0.1789 |
|  | 1.6 | 0 | primary |  | both | others | VS | 0.2553 |
| Fuchs et al. (2018) | 1 | 0 | primary | western | intra | spot | UWM | 0.40006 |
|  | 1 | 0 | primary | western | dressed | spot | UWM | 0.53606 |
|  | 1 | 0 | primary | western | intra | spot | UWM | 0.435611 |
|  | 1 | 0 | primary | western | dressed | spot | UWM | 0.435611 |
|  | 1 | 0 | primary | western | dressed | spot | UWM | 0.376886 |
|  | 1 | 0 | primary | western | intra | sentence | UWM | 0.376886 |
|  | 1 | 0 | primary | western | dressed | sentence | UWM | 0.604156 |
|  | 1 | 0 | primary | western | intra | sentence | UWM | 0.365444 |
|  | 1 | 0 | primary | western | dressed | sentence | UWM | 0.618381 |
|  | 1 | 0 | primary | western | dressed | sentence | UWM | 0.497311 |
| Bailey et al. (2012) |  | 0 | primary |  | intra | others | CE | 0.213171 |
| Fuchs et al. (2006) | 0.91411 | 1 | primary | western | intra | others | UWM | 0.192337 |
|  | 0.91411 | 1 | primary | western | intra | others | UWM | 0.202733 |
|  | 0.91411 | 1 | primary | western | dressed | others | UWM | 0.171667 |
|  | 0.91411 | 1 | primary | western | intra | sentence | UWM | 0.266108 |
|  | 0.91411 | 1 | primary | western | intra | sentence | UWM | 0.276864 |
|  | 0.91411 | 1 | primary | western | dressed | sentence | UWM | 0.4118 |
|  | 0.91411 | 1 | primary | western | intra | digit | UWM | 0.266108 |
|  | 0.91411 | 1 | primary | western | intra | digit | UWM | 0.287682 |
|  | 0.91411 | 1 | primary | western | dressed | digit | UWM | 0.4118 |
| Chan & Ho (2010) |  | 1 | primary | eastern | intra | digit | PL | 0.320545 |
|  |  | 1 | primary | eastern | intra | digit | PL | 0.287682 |
|  |  | 1 | primary | eastern | intra | digit | PL | 0.51007 |
|  |  | 1 | primary | eastern | intra | digit | PL | 0.423649 |
|  |  | 1 | primary | eastern | intra | digit | PL | 0.354093 |
|  |  | 1 | primary | eastern | intra | others | VS | 0.234189 |
|  |  | 1 | primary | eastern | intra | others | VS | 0.140926 |
|  |  | 1 | primary | eastern | intra | others | VS | 0.140926 |
|  |  | 1 | primary | eastern | intra | others | VS | 0.223656 |
|  |  | 1 | primary | eastern | intra | others | VS | 0.213171 |
| Passolunghi et al. (2014) | 1.038961 | 0 | primary | western | intra | others | UWM | 0.287682 |
|  | 1.038961 | 0 | primary | western | intra | digit | UWM | 0.331647 |
|  | 1.038961 | 0 | primary | western | intra | others | VS | 0.213171 |
|  | 1.038961 | 0 | primary | western | intra | others | PL | 0.171667 |
|  | 1.038961 | 0 | primary | western | intra | digit | PL | 0.354093 |
|  | 1.038961 | 0 | primary | western | intra | others | PL | 0.298566 |
|  | 1.038961 | 0 | primary | western | intra | others | VS | 0.365444 |
|  | 1.038961 | 0 | primary | western | intra | others | UWM | 0.213171 |
|  | 1.038961 | 0 | primary | western | intra | digit | UWM | 0.266108 |
|  | 1.038961 | 0 | primary | western | intra | others | VS | 0.192337 |
|  | 1.038961 | 0 | primary | western | intra | others | PL | 0.070115 |
|  | 1.038961 | 0 | primary | western | intra | digit | PL | 0.181983 |
|  | 1.038961 | 0 | primary | western | intra | others | PL | 0.287682 |
|  | 1.038961 | 0 | primary | western | intra | others | VS | 0.266108 |
|  | 1.038961 | 0 | primary | western | intra | others | UWM | 0.276864 |
|  | 1.038961 | 0 | primary | western | intra | digit | UWM | 0.320545 |
|  | 1.038961 | 0 | primary | western | intra | others | VS | 0.223656 |
|  | 1.038961 | 0 | primary | western | intra | others | PL | 0.192337 |
|  | 1.038961 | 0 | primary | western | intra | digit | PL | 0.354093 |
|  | 1.038961 | 0 | primary | western | intra | others | PL | 0.255413 |
|  | 1.038961 | 0 | primary | western | intra | others | VS | 0.331647 |
|  | 1.038961 | 0 | primary | western | intra | others | UWM | 0.202733 |
|  | 1.038961 | 0 | primary | western | intra | digit | UWM | 0.244774 |
|  | 1.038961 | 0 | primary | western | intra | others | VS | -0.01 |
|  | 1.038961 | 0 | primary | western | intra | others | PL | 0.020003 |
|  | 1.038961 | 0 | primary | western | intra | digit | PL | -0.040021 |
|  | 1.038961 | 0 | primary | western | intra | others | PL | 0.070115 |
|  | 1.038961 | 0 | primary | western | intra | others | VS | -0.030009 |
|  | 1.038961 | 0 | primary | western | intra | others | UWM | 0.140926 |
|  | 1.038961 | 0 | primary | western | intra | digit | UWM | -0.020003 |
|  | 1.038961 | 0 | primary | western | intra | others | VS | -0.030009 |
|  | 1.038961 | 0 | primary | western | intra | others | PL | 0.040021 |
|  | 1.038961 | 0 | primary | western | intra | digit | PL | -0.080171 |
|  | 1.038961 | 0 | primary | western | intra | others | PL | 0.050042 |
|  | 1.038961 | 0 | primary | western | intra | others | UWM | 0.161387 |
|  | 1.038961 | 0 | primary | western | intra | digit | UWM | 0.030009 |
|  | 1.038961 | 0 | primary | western | intra | others | VS | 0.030009 |
|  | 1.038961 | 0 | primary | western | intra | others | PL | 0.255413 |
|  | 1.038961 | 0 | primary | western | intra | digit | PL | 0.202733 |
|  | 1.038961 | 0 | primary | western | intra | others | PL | 0.15114 |
|  | 1.038961 | 0 | primary | western | intra | others | VS | 0.298566 |
| Traff (2013) | 0.763158 | 0 | primary | western | intra | spot | VS | 0.40006 |
|  | 0.763158 | 0 | primary | western | intra | spot | VS | 0.40006 |
|  | 0.763158 | 0 | primary | western | dressed | spot | VS | 0.388423 |
|  | 0.763158 | 0 | primary | western | intra | spot | VS | 0.331647 |
|  | 0.763158 | 0 | primary | western | intra | spot | VS | 0.244774 |
|  | 0.763158 | 0 | primary | western | intra | spot | VS | 0.223656 |
|  | 0.763158 | 0 | primary | western | intra | sentence | PL | 0.30952 |
|  | 0.763158 | 0 | primary | western | intra | sentence | PL | 0.276864 |
|  | 0.763158 | 0 | primary | western | dressed | sentence | PL | 0.255413 |
|  | 0.763158 | 0 | primary | western | intra | sentence | PL | 0.244774 |
|  | 0.763158 | 0 | primary | western | intra | sentence | PL | 0.202733 |
|  | 0.763158 | 0 | primary | western | intra | sentence | PL | 0.255413 |
|  | 0.763158 | 0 | primary | western | intra | others | UWM | 0.447692 |
|  | 0.763158 | 0 | primary | western | intra | others | UWM | 0.549306 |
|  | 0.763158 | 0 | primary | western | dressed | others | UWM | 0.57634 |
|  | 0.763158 | 0 | primary | western | intra | others | UWM | 0.435611 |
|  | 0.763158 | 0 | primary | western | intra | others | UWM | 0.331647 |
|  | 0.763158 | 0 | primary | western | intra | others | UWM | 0.331647 |
|  | 0.763158 | 0 | primary | western | intra | others | UWM | 0.522984 |
|  | 0.763158 | 0 | primary | western | intra | others | UWM | 0.388423 |
|  | 0.763158 | 0 | primary | western | dressed | others | UWM | 0.435611 |
|  | 0.763158 | 0 | primary | western | intra | others | UWM | 0.51007 |
|  | 0.763158 | 0 | primary | western | intra | others | UWM | 0.192337 |
|  | 0.763158 | 0 | primary | western | intra | others | UWM | 0.4847 |
| Trakulphadetkrai et al. (2020) | 1 | 0 | primary | western | both | digit | UWM | 0.295294 |
| Anderisson (2007) | 1.090909 | 0 | primary | western | dressed | spot | CE | 0.365444 |
|  | 1.090909 | 0 | primary | western | intra | spot | CE | 0.266108 |
|  | 1.090909 | 0 | primary | western | dressed | others | CE | 0.13074 |
|  | 1.090909 | 0 | primary | western | intra | others | CE | 0.298566 |
|  | 1.090909 | 0 | primary | western | dressed | others | CE | 0.472231 |
|  | 1.090909 | 0 | primary | western | intra | others | CE | 0.376886 |
|  | 1.090909 | 0 | primary | western | dressed | others | CE | 0.604156 |
|  | 1.090909 | 0 | primary | western | intra | others | CE | 0.775299 |
|  | 1.090909 | 0 | primary | western | dressed | digit | PL | 0.354093 |
|  | 1.090909 | 0 | primary | western | intra | digit | PL | 0.331647 |
| Lee et al. (2009) | 1.073171 | 0 | primary | eastern | dressed | spot | UWM | 0.343951 |
|  | 1.073171 | 0 | primary | eastern | dressed | spot | UWM | 0.323868 |
|  | 1.073171 | 0 | primary | eastern | dressed | spot | UWM | 0.222605 |
|  | 1.073171 | 0 | primary | eastern | dressed | spot | UWM | 0.318334 |
|  | 1.073171 | 0 | primary | eastern | dressed | spot | UWM | 0.260753 |
|  | 1.073171 | 0 | primary | eastern | intra | spot | UWM | 0.249023 |
| Imbo &Vandierendonck (2007) | 0.909091 | 0 | primary | western | intra | digit | UWM | 0.140926 |
| Swanson et al. (2014) |  | 1 | primary | western | dressed | others | UWM | 0.604156 |
|  |  | 1 | primary | western | dressed | others | UWM | -0.181983 |
|  |  | 1 | primary | western | dressed | others | UWM | -0.388423 |
|  |  | 1 | primary | western | dressed | others | UWM | -0.907645 |
|  |  | 1 | primary | western | dressed | others | UWM | 0.792814 |
| Ching & Nunes 2017 | 1.12963 | 0 | primary | eastern | intra | digit | CE | 0.15114 |
|  | 1.12963 | 0 | primary | eastern | intra | digit | CE | 0.13074 |
|  | 1.12963 | 0 | primary | eastern | intra | digit | CE | 0.100335 |
|  | 1.12963 | 0 | primary | eastern | intra | digit | CE | 0.140926 |
|  | 1.12963 | 0 | primary | eastern | intra | digit | CE | 0.365444 |
|  | 1.12963 | 0 | primary | eastern | intra | digit | CE | 0.447692 |
|  | 1.12963 | 0 | primary | eastern | dressed | digit | CE | 0.342828 |
|  | 1.12963 | 0 | primary | eastern | dressed | digit | CE | 0.365444 |
|  | 1.12963 | 0 | primary | eastern | intra | digit | PL | 0.181983 |
|  | 1.12963 | 0 | primary | eastern | intra | digit | PL | 0.202733 |
|  | 1.12963 | 0 | primary | eastern | intra | digit | PL | 0.140926 |
|  | 1.12963 | 0 | primary | eastern | intra | digit | PL | 0.020003 |
|  | 1.12963 | 0 | primary | eastern | intra | digit | PL | 0.192337 |
|  | 1.12963 | 0 | primary | eastern | intra | digit | PL | 0.255413 |
|  | 1.12963 | 0 | primary | eastern | dressed | digit | PL | 0.040021 |
|  | 1.12963 | 0 | primary | eastern | dressed | digit | PL | 0.100335 |
|  | 1.12963 | 0 | primary | eastern | intra | block | VS | 0.13074 |
|  | 1.12963 | 0 | primary | eastern | intra | block | VS | 0.120581 |
|  | 1.12963 | 0 | primary | eastern | intra | block | VS | 0.040021 |
|  | 1.12963 | 0 | primary | eastern | intra | block | VS | 0.030009 |
|  | 1.12963 | 0 | primary | eastern | intra | block | VS | 0.060072 |
|  | 1.12963 | 0 | primary | eastern | intra | block | VS | 0.140926 |
|  | 1.12963 | 0 | primary | eastern | dressed | block | VS | 0.040021 |
|  | 1.12963 | 0 | primary | eastern | dressed | block | VS | 0.050042 |
| Jordan et al. (2010) | 1.222222 | 0 | primary | western | intra | digit | UWM | -0.040021 |
|  | 1.222222 | 0 | primary | western | intra | digit | UWM | 0.234189 |
|  | 1.173913 | 0 | primary | western | intra | digit | UWM | -0.020003 |
|  | 1.173913 | 0 | primary | western | intra | digit | UWM | 0.080171 |
|  | 1.222222 | 0 | primary | western | dressed | digit | UWM | 0.13074 |
|  | 1.222222 | 0 | primary | western | dressed | digit | UWM | 0.100335 |
|  | 1.173913 | 0 | primary | western | dressed | digit | UWM | 0.100335 |
|  | 1.173913 | 0 | primary | western | dressed | digit | UWM | 0.060072 |
| Bresgi et al. (2017) | 1.222222 | 0 | primary |  |  | sentence | PL | 0.366584 |
|  | 1.222222 | 0 | primary |  |  | sentence | PL | 0.347324 |
|  | 1.222222 | 0 | primary |  |  | others | VS | 0.457446 |
|  | 1.222222 | 0 | primary |  |  | others | VS | 0.479696 |
| Passolunghi et al. (2019) | 1.042254 | 0 | primary | western | dressed | sentence | UWM | 0.266108 |
|  | 1.042254 | 0 | primary | western | dressed | sentence | UWM | 0.171667 |
|  | 1.042254 | 0 | primary | western | dressed | sentence | UWM | 0.244774 |
|  | 1.042254 | 0 | primary | western | dressed | others | UWM | 0.181983 |
|  | 1.042254 | 0 | primary | western | dressed | digit | UWM | 0.234189 |
| Tam et al. (2019) | 1.073171 | 0 | primary | eastern | intra | others | VS | 0.219457 |
|  | 1.073171 | 0 | primary | eastern | intra | others | VS | 0.177852 |
|  | 1.073171 | 0 | primary | eastern | intra | others | VS | 0.237359 |
|  | 1.073171 | 0 | primary | eastern | dressed | others | VS | 0.326087 |
| Hecht et al. (2001) | 0.851852 | 0 | primary | western | intra | others | PL | 0.388423 |
|  | 0.851852 | 0 | primary | western | intra | others | PL | 0.447692 |
|  | 0.851852 | 0 | primary | western | intra | others | PL | 0.435611 |
|  | 0.851852 | 0 | primary | western | intra | others | PL | 0.388423 |
| Giofre et al. (2014) | 1.2 | 0 | primary | western | intra | others | PL | 0.266108 |
|  | 1.2 | 0 | primary | western | intra | others | PL | 0.223656 |
|  | 1.2 | 0 | primary | western | intra | block | VS | 0.213171 |
|  | 1.2 | 0 | primary | western | intra | block | VS | 0.171667 |
|  | 1.2 | 0 | primary | western | intra | digit | UWM | 0.276864 |
|  | 1.2 | 0 | primary | western | intra | sentence | UWM | 0.298566 |
|  | 1.2 | 0 | primary | western | intra | block | UWM | 0.213171 |
|  | 1.2 | 0 | primary | western | intra | others | PL | 0.266108 |
|  | 1.2 | 0 | primary | western | intra | others | PL | 0.181983 |
|  | 1.2 | 0 | primary | western | intra | block | VS | 0.100335 |
|  | 1.2 | 0 | primary | western | intra | block | VS | 0.161387 |
|  | 1.2 | 0 | primary | western | intra | digit | UWM | 0.255413 |
|  | 1.2 | 0 | primary | western | intra | sentence | UWM | 0.287682 |
|  | 1.2 | 0 | primary | western | intra | block | UWM | 0.266108 |
|  | 1.2 | 0 | primary | western | intra | others | PL | 0.255413 |
|  | 1.2 | 0 | primary | western | intra | others | PL | 0.213171 |
|  | 1.2 | 0 | primary | western | intra | block | VS | 0.331647 |
|  | 1.2 | 0 | primary | western | intra | block | VS | 0.40006 |
|  | 1.2 | 0 | primary | western | intra | digit | UWM | 0.331647 |
|  | 1.2 | 0 | primary | western | intra | sentence | UWM | 0.223656 |
|  | 1.2 | 0 | primary | western | intra | block | UWM | 0.388423 |
| Tavakoli (2016) |  | 0 | primary |  | intra | others | UWM | 0.181983 |
|  |  | 0 | primary |  | intra | others | UWM | 0.171667 |
|  |  | 0 | primary |  | intra | others | UWM | 0.223656 |
|  |  | 0 | primary |  | intra | others | UWM | 0.171667 |
|  |  | 0 | primary |  | intra | others | UWM | 0.161387 |
|  |  | 0 | primary |  | intra | others | UWM | 0.140926 |
|  |  | 0 | primary |  | intra | spot | UWM | 0.4118 |
|  |  | 0 | primary |  | intra | spot | UWM | 0.320545 |
|  |  | 0 | primary |  | intra | spot | UWM | 0.459897 |
|  |  | 0 | primary |  | intra | spot | UWM | 0.354093 |
|  |  | 0 | primary |  | intra | spot | UWM | 0.266108 |
|  |  | 0 | primary |  | intra | spot | UWM | 0.320545 |
| Swanson & Beebe-Frankenberger (2004) | 1.088757 | 1 | primary | western | dressed | others | UWM | 0.51007 |
|  | 1.088757 | 1 | primary | western | intra | others | UWM | 0.423649 |
|  | 1.088757 | 1 | primary | western | intra | others | UWM | 0.331647 |
|  | 1.088757 | 1 | primary | western | dressed | others | UWM | 0.604156 |
|  | 1.088757 | 1 | primary | western | intra | others | UWM | 0.56273 |
|  | 1.088757 | 1 | primary | western | intra | others | UWM | 0.365444 |
| Attout et al. (2014) |  | 0 | primary | western | intra | others | UWM | 0.388423 |
|  |  | 0 | primary | western | intra | others | UWM | 0.459897 |
|  |  | 0 | primary | western | intra | others | UWM | 0.266108 |
|  |  | 0 | primary | western | intra | others | UWM | 0.223656 |
|  |  | 0 | primary | western | intra | others | UWM | 0.276864 |
|  |  | 0 | primary | western | intra | others | UWM | 0.51007 |
| Nunes et al. (2012) |  | 0 | primary | western | dressed | digit | UWM | 0.287682 |
|  |  | 0 | primary | western | intra | digit | UWM | 0.331647 |
|  | 0.923077 | 0 | primary | western | both | digit | UWM | 0.342828 |
|  | 0.886792 | 0 | primary | western | both | digit | UWM | 0.354093 |
| Huijsmans et al. (2020) | 1 | 0 | primary | western | intra | others | UWM | 0.192337 |
|  | 1 | 0 | primary | western | dressed | others | UWM | 0.40006 |
| Fuchs et al. (2016) | 1.083333 | 0 | primary | western | dressed | sentence | UWM | 0.459897 |
|  | 1.083333 | 0 | primary | western | intra | sentence | UWM | 0.423649 |
|  | 1.083333 | 0 | primary | western | intra | sentence | UWM | 0.234189 |
|  | 1.083333 | 0 | primary | western | intra | sentence | UWM | 0.192337 |
|  | 1.083333 | 0 | primary | western | intra | sentence | UWM | 0.276864 |
|  | 1.083333 | 0 | primary | western | dressed | sentence | UWM | 0.472231 |
|  | 1.083333 | 0 | primary | western | intra | sentence | UWM | 0.266108 |
| LeFevre et al. (2013) | 0.779661 | 0 | primary |  | intra | others | CE | 0.140926 |
|  | 0.779661 | 0 | primary |  | intra | others | CE | 0.15114 |
|  | 0.779661 | 0 | primary |  | intra | others | CE | 0.110447 |
|  | 0.779661 | 0 | primary |  | intra | others | CE | 0.331647 |
|  | 0.779661 | 0 | primary |  | intra | others | CE | 0.388423 |
|  | 0.779661 | 0 | primary |  | intra | digit | CE | 0.161387 |
|  | 0.779661 | 0 | primary |  | intra | digit | CE | 0.15114 |
|  | 0.779661 | 0 | primary |  | intra | digit | CE | 0.080171 |
|  | 0.779661 | 0 | primary |  | intra | digit | CE | 0.161387 |
|  | 0.779661 | 0 | primary |  | intra | digit | CE | 0.213171 |
|  | 0.779661 | 0 | primary |  | intra | others | CE | 0.320545 |
|  | 0.779661 | 0 | primary |  | intra | others | CE | 0.266108 |
|  | 0.779661 | 0 | primary |  | intra | others | CE | 0.30952 |
|  | 0.779661 | 0 | primary |  | intra | others | CE | 0.202733 |
|  | 0.779661 | 0 | primary |  | intra | others | CE | 0.342828 |
|  | 0.833333 | 0 | primary |  | intra | others | CE | 0.223656 |
|  | 0.833333 | 0 | primary |  | intra | others | CE | 0.255413 |
|  | 0.833333 | 0 | primary |  | intra | others | CE | 0.287682 |
|  | 0.833333 | 0 | primary |  | intra | others | CE | 0.181983 |
|  | 0.833333 | 0 | primary |  | intra | others | CE | 0.181983 |
|  | 0.833333 | 0 | primary |  | intra | others | CE | 0.202733 |
|  | 0.833333 | 0 | primary |  | intra | digit | CE | 0.287682 |
|  | 0.833333 | 0 | primary |  | intra | digit | CE | 0.276864 |
|  | 0.833333 | 0 | primary |  | intra | digit | CE | 0.287682 |
|  | 0.833333 | 0 | primary |  | intra | digit | CE | 0.266108 |
|  | 0.833333 | 0 | primary |  | intra | digit | CE | 0.161387 |
|  | 0.833333 | 0 | primary |  | intra | digit | CE | 0.223656 |
|  | 0.833333 | 0 | primary |  | intra | others | CE | 0.331647 |
|  | 0.833333 | 0 | primary |  | intra | others | CE | 0.298566 |
|  | 0.833333 | 0 | primary |  | intra | others | CE | 0.40006 |
|  | 0.833333 | 0 | primary |  | intra | others | CE | 0.287682 |
|  | 0.833333 | 0 | primary |  | intra | others | CE | 0.181983 |
|  | 0.833333 | 0 | primary |  | intra | others | CE | 0.15114 |
| Viterbori et al. (2017) | 1.325581 | 0 | primary | western | dressed | others | CE | 0.2407 |
|  | 1.325581 | 0 | primary | western | intra | others | CE | -0.20586 |
| Cai et al. (2016) | 1.352941 | 0 | primary | eastern | intra | digit | UWM | 0.202733 |
|  | 1.352941 | 0 | primary | eastern | dressed | digit | UWM | 0.388423 |
|  | 1.352941 | 0 | primary | eastern | intra | digit | UWM | 0.234189 |
|  | 1.352941 | 0 | primary | eastern | intra | others | UWM | 0.244774 |
|  | 1.352941 | 0 | primary | eastern | dressed | others | UWM | 0.447692 |
|  | 1.352941 | 0 | primary | eastern | intra | others | UWM | 0.30952 |
| Iglesias-Sarmiento et al. (2015) | 0.484848 | 0 | primary | western | dressed | others | CE | 0.522984 |
|  | 0.484848 | 0 | primary | western | intra | others | CE | 0.354093 |
| Reeve et al. (2018) | 1.523077 | 0 | primary | western | intra | block | VS | 0.040021 |
|  | 1.523077 | 0 | primary | western | intra | digit | PL | 0.120581 |
| Fanari et al. (2019) | 1.15 | 0 | primary | western | intra | others | VS | 0.376886 |
|  | 1.15 | 0 | primary | western | intra | others | VS | 0.423649 |
|  | 1.15 | 0 | primary | western | intra | others | VS | 0.050042 |
|  | 1.15 | 0 | primary | western | intra | block | VS | 0.497311 |
|  | 1.15 | 0 | primary | western | intra | block | VS | 0.365444 |
|  | 1.15 | 0 | primary | western | intra | block | VS | 0.181983 |
|  | 1.15 | 0 | primary | western | intra | others | VS | 0.57634 |
|  | 1.15 | 0 | primary | western | intra | others | VS | 0.320545 |
|  | 1.15 | 0 | primary | western | intra | others | VS | 0.15114 |
|  | 1.15 | 0 | primary | western | intra | block | VS | 0.365444 |
|  | 1.15 | 0 | primary | western | intra | block | VS | 0.234189 |
|  | 1.15 | 0 | primary | western | intra | block | VS | 0.30952 |
|  | 1.15 | 0 | primary | western | intra | others | VS | 0.298566 |
|  | 1.15 | 0 | primary | western | intra | others | VS | 0.287682 |
|  | 1.15 | 0 | primary | western | intra | others | VS | 0.331647 |
|  | 1.15 | 0 | primary | western | intra | block | VS | 0.266108 |
|  | 1.15 | 0 | primary | western | intra | block | VS | 0.213171 |
|  | 1.15 | 0 | primary | western | intra | block | VS | 0.40006 |
| Ashkenazi et al. (2013) | 0.545455 | 1 | primary | western | intra | digit | PL | -0.090244 |
|  | 0.545455 | 1 | primary | western | both | digit | PL | 0.050042 |
|  | 0.545455 | 1 | primary | western | intra | spot | CE | 0.140926 |
|  | 0.545455 | 1 | primary | western | both | spot | CE | 0.298566 |
|  | 0.545455 | 1 | primary | western | intra | block | VS | 0.497311 |
|  | 0.545455 | 1 | primary | western | both | block | VS | 0.202733 |
|  | 0.545455 | 1 | primary | western | intra | digit | CE | 0.13074 |
|  | 0.545455 | 1 | primary | western | both | digit | CE | 0.171667 |
| Chan & Wang (2019) | 1.318367 | 0 | primary | eastern | both | digit | PL | 0.320545 |
|  | 1.318367 | 0 | primary | eastern | intra | digit | PL | 0.140926 |
|  | 1.318367 | 0 | primary | eastern | intra | digit | PL | 0.181983 |
|  | 1.318367 | 0 | primary | eastern | dressed | digit | PL | 0.266108 |
|  | 1.302128 | 0 | primary | eastern | both | digit | PL | 0.266108 |
|  | 1.318367 | 0 | primary | eastern | both | block | VS | 0.320545 |
|  | 1.318367 | 0 | primary | eastern | intra | block | VS | 0.223656 |
|  | 1.318367 | 0 | primary | eastern | intra | block | VS | 0.202733 |
|  | 1.318367 | 0 | primary | eastern | dressed | block | VS | 0.320545 |
|  | 1.302128 | 0 | primary | eastern | both | block | VS | 0.342828 |
| Berg (2008) | 0.956522 | 0 | primary | western | intra | digit | UWM | 0.647523 |
|  | 0.956522 | 0 | primary | western | intra | others | UWM | 0.459897 |
|  | 0.956522 | 0 | primary | western | intra | block | VS | 0.497311 |
|  | 0.956522 | 0 | primary | western | intra | spot | VS | 0.472231 |
|  | 0.956522 | 0 | primary | western | intra | others | PL | 0.57634 |
|  | 0.956522 | 0 | primary | western | intra | others | PL | 0.331647 |
| Caviola et al. (2014) | 1.833333 | 0 | primary | western | intra | block | UWM | 0.161387 |
|  | 1.833333 | 0 | primary | western | intra | block | UWM | 0.255413 |
|  | 1.833333 | 0 | primary | western | intra | block | UWM | 0.110447 |
|  | 1.833333 | 0 | primary | western | intra | block | UWM | 0.171667 |
|  | 1.833333 | 0 | primary | western | intra | block | UWM | -0.171667 |
|  | 1.833333 | 0 | primary | western | intra | block | UWM | -0.040021 |
|  | 1.833333 | 0 | primary | western | intra | block | UWM | 0.298566 |
|  | 1.833333 | 0 | primary | western | intra | block | UWM | 0.080171 |
|  | 1.833333 | 0 | primary | western | intra | block | UWM | 0.320545 |
|  | 1.833333 | 0 | primary | western | intra | block | UWM | -0.080171 |
|  | 1.833333 | 0 | primary | western | intra | block | UWM | 0.110447 |
|  | 1.833333 | 0 | primary | western | intra | block | UWM | 0.266108 |
|  | 1.833333 | 0 | primary | western | intra | others | UWM | 0.266108 |
|  | 1.833333 | 0 | primary | western | intra | others | UWM | 0.15114 |
|  | 1.833333 | 0 | primary | western | intra | others | UWM | 0.266108 |
|  | 1.833333 | 0 | primary | western | intra | others | UWM | 0.202733 |
|  | 1.833333 | 0 | primary | western | intra | others | UWM | 0.181983 |
|  | 1.833333 | 0 | primary | western | intra | others | UWM | 0.181983 |
|  | 1.833333 | 0 | primary | western | intra | others | UWM | 0.050042 |
|  | 1.833333 | 0 | primary | western | intra | others | UWM | 0.030009 |
|  | 1.833333 | 0 | primary | western | intra | others | UWM | 0.060072 |
|  | 1.833333 | 0 | primary | western | intra | others | UWM | 0.161387 |
|  | 1.833333 | 0 | primary | western | intra | others | UWM | 0.040021 |
|  | 1.833333 | 0 | primary | western | intra | others | UWM | 0.070115 |
|  | 1.833333 | 0 | primary | western | intra | spot | UWM | 0.447692 |
|  | 1.833333 | 0 | primary | western | intra | spot | UWM | 0.331647 |
|  | 1.833333 | 0 | primary | western | intra | spot | UWM | 0.202733 |
|  | 1.833333 | 0 | primary | western | intra | spot | UWM | 0.40006 |
|  | 1.833333 | 0 | primary | western | intra | spot | UWM | 0.140926 |
|  | 1.833333 | 0 | primary | western | intra | spot | UWM | 0.234189 |
|  | 1.833333 | 0 | primary | western | intra | spot | UWM | 0.423649 |
|  | 1.833333 | 0 | primary | western | intra | spot | UWM | 0.15114 |
|  | 1.833333 | 0 | primary | western | intra | spot | UWM | 0.53606 |
|  | 1.833333 | 0 | primary | western | intra | spot | UWM | 0.020003 |
|  | 1.833333 | 0 | primary | western | intra | spot | UWM | 0.423649 |
|  | 1.833333 | 0 | primary | western | intra | spot | UWM | 0.40006 |
|  | 1.833333 | 0 | primary | western | intra | others | UWM | 0.140926 |
|  | 1.833333 | 0 | primary | western | intra | others | UWM | 0.140926 |
|  | 1.833333 | 0 | primary | western | intra | others | UWM | 0.15114 |
|  | 1.833333 | 0 | primary | western | intra | others | UWM | 0.192337 |
|  | 1.833333 | 0 | primary | western | intra | others | UWM | 0.040021 |
|  | 1.833333 | 0 | primary | western | intra | others | UWM | 0.30952 |
|  | 1.833333 | 0 | primary | western | intra | others | UWM | 0.181983 |
|  | 1.833333 | 0 | primary | western | intra | others | UWM | 0.13074 |
|  | 1.833333 | 0 | primary | western | intra | others | UWM | 0.255413 |
|  | 1.833333 | 0 | primary | western | intra | others | UWM | 0.090244 |
|  | 1.833333 | 0 | primary | western | intra | others | UWM | 0.140926 |
|  | 1.833333 | 0 | primary | western | intra | others | UWM | 0.223656 |
| Lee et al. (2004) | 1.040541 | 0 | primary | eastern | dressed | digit | PL | 0.388423 |
|  | 1.040541 | 0 | primary | eastern | dressed | others | VS | 0.417711 |
|  | 1.040541 | 0 | primary | eastern | dressed | spot | CE | 0.572237 |
| Swanson (2004) | 2 | 0 | primary | western | dressed | others | UWM | 0.632833 |
|  | 2 | 0 | primary | western | dressed | others | UWM | 0.120581 |
|  | 2 | 0 | primary | western | intra | others | UWM | -0.050042 |
|  | 2 | 0 | primary | western | dressed | others | UWM | 0.266108 |
|  | 2 | 0 | primary | western | dressed | others | UWM | 0.276864 |
|  | 2 | 0 | primary | western | intra | others | UWM | 0.354093 |
|  | 2 | 0 | primary | western | dressed | others | UWM | 0.030009 |
|  | 2 | 0 | primary | western | intra | others | UWM | 0.459897 |
| Andersson (2008) | 0.698795 | 0 | primary | western | intra | spot | CE | 0.618381 |
|  | 0.698795 | 0 | primary | western | intra | spot | CE | 0.4847 |
|  | 0.698795 | 0 | primary | western | intra | spot | CE | 0.604156 |
|  | 0.698795 | 0 | primary | western | intra | spot | CE | 0.549306 |
|  | 0.698795 | 0 | primary | western | intra | spot | CE | 0.388423 |
|  | 0.698795 | 0 | primary | western | intra | spot | CE | 0.549306 |
|  | 0.698795 | 0 | primary | western | intra | spot | CE | 0.435611 |
|  | 0.698795 | 0 | primary | western | intra | spot | CE | 0.57634 |
|  | 0.698795 | 0 | primary | western | intra | spot | CE | 0.447692 |
|  | 0.698795 | 0 | primary | western | intra | spot | CE | 0.472231 |
|  | 0.698795 | 0 | primary | western | intra | others | CE | 0.708921 |
|  | 0.698795 | 0 | primary | western | intra | others | CE | 0.57634 |
|  | 0.698795 | 0 | primary | western | intra | others | CE | 0.662463 |
|  | 0.698795 | 0 | primary | western | intra | others | CE | 0.618381 |
|  | 0.698795 | 0 | primary | western | intra | others | CE | 0.618381 |
|  | 0.698795 | 0 | primary | western | intra | others | CE | 0.287682 |
|  | 0.698795 | 0 | primary | western | intra | others | CE | 0.234189 |
|  | 0.698795 | 0 | primary | western | intra | others | CE | 0.244774 |
|  | 0.698795 | 0 | primary | western | intra | others | CE | 0.298566 |
|  | 0.698795 | 0 | primary | western | intra | others | CE | 0.298566 |
|  | 0.698795 | 0 | primary | western | intra | digit | PL | 0.497311 |
|  | 0.698795 | 0 | primary | western | intra | digit | PL | 0.423649 |
|  | 0.698795 | 0 | primary | western | intra | digit | PL | 0.423649 |
|  | 0.698795 | 0 | primary | western | intra | digit | PL | 0.459897 |
|  | 0.698795 | 0 | primary | western | intra | digit | PL | 0.354093 |
|  | 0.698795 | 0 | primary | western | intra | block | VS | 0.342828 |
|  | 0.698795 | 0 | primary | western | intra | block | VS | 0.266108 |
|  | 0.698795 | 0 | primary | western | intra | block | VS | 0.354093 |
|  | 0.698795 | 0 | primary | western | intra | block | VS | 0.287682 |
|  | 0.698795 | 0 | primary | western | intra | block | VS | 0.40006 |
| Zheng et al. (2011) | 0.925466 | 0 | primary | western | dressed | spot | VS | 0.4847 |
|  | 0.925466 | 0 | primary | western | dressed | spot | VS | 0.244774 |
|  | 0.925466 | 0 | primary | western | dressed | spot | VS | 0.40006 |
|  | 0.925466 | 0 | primary | western | dressed | spot | VS | 0.40006 |
|  | 0.925466 | 0 | primary | western | intra | spot | VS | 0.266108 |
|  | 0.925466 | 0 | primary | western | intra | spot | VS | 0.298566 |
|  | 0.925466 | 0 | primary | western | dressed | others | VS | 0.447692 |
|  | 0.925466 | 0 | primary | western | dressed | others | VS | 0.244774 |
|  | 0.925466 | 0 | primary | western | dressed | others | VS | 0.40006 |
|  | 0.925466 | 0 | primary | western | dressed | others | VS | 0.497311 |
|  | 0.925466 | 0 | primary | western | intra | others | VS | 0.234189 |
|  | 0.925466 | 0 | primary | western | intra | others | VS | 0.2447741 |
|  | 0.925466 | 0 | primary | western | dressed | others | PL | 0.276864 |
|  | 0.925466 | 0 | primary | western | dressed | others | PL | 0.202733 |
|  | 0.925466 | 0 | primary | western | dressed | others | PL | 0.223656 |
|  | 0.925466 | 0 | primary | western | dressed | others | PL | 0.244774 |
|  | 0.925466 | 0 | primary | western | intra | others | PL | 0.320545 |
|  | 0.925466 | 0 | primary | western | intra | others | PL | 0.342828 |
|  | 0.925466 | 0 | primary | western | dressed | sentence | CE | 0.320545 |
|  | 0.925466 | 0 | primary | western | dressed | sentence | CE | 0.202733 |
|  | 0.925466 | 0 | primary | western | dressed | sentence | CE | 0.202733 |
|  | 0.925466 | 0 | primary | western | dressed | sentence | CE | 0.244774 |
|  | 0.925466 | 0 | primary | western | intra | sentence | CE | 0.4118 |
|  | 0.925466 | 0 | primary | western | intra | sentence | CE | 0.40006 |
|  | 0.925466 | 0 | primary | western | dressed | others | PL | 0.320545 |
|  | 0.925466 | 0 | primary | western | dressed | others | PL | 0.15114 |
|  | 0.925466 | 0 | primary | western | dressed | others | PL | 0.354093 |
|  | 0.925466 | 0 | primary | western | dressed | others | PL | 0.342828 |
|  | 0.925466 | 0 | primary | western | intra | others | PL | 0.298566 |
|  | 0.925466 | 0 | primary | western | intra | others | PL | 0.298566 |
|  | 0.925466 | 0 | primary | western | dressed | others | CE | 0.244774 |
|  | 0.925466 | 0 | primary | western | dressed | others | CE | 0.202733 |
|  | 0.925466 | 0 | primary | western | dressed | others | CE | 0.331647 |
|  | 0.925466 | 0 | primary | western | dressed | others | CE | 0.287682 |
|  | 0.925466 | 0 | primary | western | intra | others | CE | 0.320545 |
|  | 0.925466 | 0 | primary | western | intra | others | CE | 0.30952 |
|  | 0.925466 | 0 | primary | western | dressed | others | PL | 0.320545 |
|  | 0.925466 | 0 | primary | western | dressed | others | PL | 0.140926 |
|  | 0.925466 | 0 | primary | western | dressed | others | PL | 0.376886 |
|  | 0.925466 | 0 | primary | western | dressed | others | PL | 0.388423 |
|  | 0.925466 | 0 | primary | western | intra | others | PL | 0.213171 |
|  | 0.925466 | 0 | primary | western | intra | others | PL | 0.234189 |
| Andersson & Lyxell (2007) |  | 1 | primary | western | intra | others | CE | -0.100335 |
|  |  | 1 | primary | western | intra | spot | CE | 0.01 |
|  |  | 1 | primary | western | intra | spot | CE | 0.376886 |
|  |  | 1 | primary | western | intra | others | CE | 0.447692 |
|  |  | 1 | primary | western | intra | others | CE | 0.549306 |
|  |  | 1 | primary | western | intra | others | CE | 0.549306 |
|  |  | 1 | primary | western | intra | others | CE | 0.522984 |
|  |  | 1 | primary | western | intra | others | CE | 0.447692 |
|  |  | 1 | primary | western | intra | digit | CE | 0.472231 |
|  |  | 1 | primary | western | intra | others | PL | -0.140926 |
|  |  | 1 | primary | western | intra | digit | PL | 0.4118 |
|  |  | 1 | primary | western | intra | block | PL | 0.140926 |
|  |  | 1 | primary | western | intra | others | CE | -0.13074 |
|  |  | 1 | primary | western | intra | spot | CE | -0.244774 |
|  |  | 1 | primary | western | intra | spot | CE | 0.120581 |
|  |  | 1 | primary | western | intra | others | CE | 0.376886 |
|  |  | 1 | primary | western | intra | others | CE | 0.51007 |
|  |  | 1 | primary | western | intra | others | CE | 0.435611 |
|  |  | 1 | primary | western | intra | others | CE | 0.30952 |
|  |  | 1 | primary | western | intra | others | CE | 0.298566 |
|  |  | 1 | primary | western | intra | digit | CE | 0.497311 |
|  |  | 1 | primary | western | intra | others | PL | -0.060072 |
|  |  | 1 | primary | western | intra | digit | PL | 0.090244 |
|  |  | 1 | primary | western | intra | block | PL | 0.354093 |
|  |  | 1 | primary | western | intra | others | CE | 0.181983 |
|  |  | 1 | primary | western | intra | spot | CE | 0.298566 |
|  |  | 1 | primary | western | intra | spot | CE | 0.549306 |
|  |  | 1 | primary | western | intra | others | CE | 0.4847 |
|  |  | 1 | primary | western | intra | others | CE | 0.213171 |
|  |  | 1 | primary | western | intra | others | CE | 0.662463 |
|  |  | 1 | primary | western | intra | others | CE | 0.388423 |
|  |  | 1 | primary | western | intra | others | CE | 0.365444 |
|  |  | 1 | primary | western | intra | digit | CE | 0.51007 |
|  |  | 1 | primary | western | intra | others | PL | 0.266108 |
|  |  | 1 | primary | western | intra | digit | PL | 0.080171 |
|  |  | 1 | primary | western | intra | block | PL | 0.287682 |
|  |  | 1 | primary | western | intra | others | CE | 0.202733 |
|  |  | 1 | primary | western | intra | spot | CE | 0.080171 |
|  |  | 1 | primary | western | intra | spot | CE | 0.459897 |
|  |  | 1 | primary | western | intra | others | CE | 0.4118 |
|  |  | 1 | primary | western | intra | others | CE | 0.202733 |
|  |  | 1 | primary | western | intra | others | CE | 0.57634 |
|  |  | 1 | primary | western | intra | others | CE | 0.376886 |
|  |  | 1 | primary | western | intra | others | CE | 0.192337 |
|  |  | 1 | primary | western | intra | digit | CE | 0.40006 |
|  |  | 1 | primary | western | intra | others | PL | 0.287682 |
|  |  | 1 | primary | western | intra | digit | PL | 0.171667 |
|  |  | 1 | primary | western | intra | block | PL | 0.423649 |
|  |  | 0 | primary | western | intra | others | CE | 0.213171 |
|  |  | 0 | primary | western | intra | spot | CE | 0.181983 |
|  |  | 0 | primary | western | intra | spot | CE | 1.256153 |
|  |  | 0 | primary | western | intra | others | CE | 0.354093 |
|  |  | 0 | primary | western | intra | others | CE | 0.775299 |
|  |  | 0 | primary | western | intra | others | CE | 0.590145 |
|  |  | 0 | primary | western | intra | others | CE | 0.234189 |
|  |  | 0 | primary | western | intra | others | CE | 0.53606 |
|  |  | 0 | primary | western | intra | digit | CE | 0.775299 |
|  |  | 0 | primary | western | intra | others | PL | -0.365444 |
|  |  | 0 | primary | western | intra | digit | PL | 0.070115 |
|  |  | 0 | primary | western | intra | block | PL | 0.472231 |
|  |  | 0 | primary | western | intra | others | CE | 0.4118 |
|  |  | 0 | primary | western | intra | spot | CE | 0.376886 |
|  |  | 0 | primary | western | intra | spot | CE | 0.435611 |
|  |  | 0 | primary | western | intra | others | CE | 0.354093 |
|  |  | 0 | primary | western | intra | others | CE | 0.140926 |
|  |  | 0 | primary | western | intra | others | CE | 0.365444 |
|  |  | 0 | primary | western | intra | others | CE | 0.549306 |
|  |  | 0 | primary | western | intra | others | CE | 0.342828 |
|  |  | 0 | primary | western | intra | digit | CE | 0.662463 |
|  |  | 0 | primary | western | intra | others | PL | 0.40006 |
|  |  | 0 | primary | western | intra | digit | PL | 0.100335 |
|  |  | 0 | primary | western | intra | block | PL | 0.522984 |
|  |  | 0 | primary | western | intra | others | CE | 0.060072 |
|  |  | 0 | primary | western | intra | spot | CE | 0.423649 |
|  |  | 0 | primary | western | intra | spot | CE | 0.100335 |
|  |  | 0 | primary | western | intra | others | CE | 0.020003 |
|  |  | 0 | primary | western | intra | others | CE | 0.181983 |
|  |  | 0 | primary | western | intra | others | CE | 0.435611 |
|  |  | 0 | primary | western | intra | others | CE | 0.234189 |
|  |  | 0 | primary | western | intra | others | CE | 0.202733 |
|  |  | 0 | primary | western | intra | digit | CE | 0.40006 |
|  |  | 0 | primary | western | intra | others | PL | 0.120581 |
|  |  | 0 | primary | western | intra | digit | PL | 0.30952 |
|  |  | 0 | primary | western | intra | block | PL | -0.030009 |
|  |  | 0 | primary | western | intra | others | CE | 0.192337 |
|  |  | 0 | primary | western | intra | spot | CE | 0.120581 |
|  |  | 0 | primary | western | intra | spot | CE | 0.100335 |
|  |  | 0 | primary | western | intra | others | CE | 0.060072 |
|  |  | 0 | primary | western | intra | others | CE | 0.15114 |
|  |  | 0 | primary | western | intra | others | CE | 0.320545 |
|  |  | 0 | primary | western | intra | others | CE | 0.244774 |
|  |  | 0 | primary | western | intra | others | CE | 0.320545 |
|  |  | 0 | primary | western | intra | digit | CE | 0.472231 |
|  |  | 0 | primary | western | intra | others | PL | 0.070115 |
|  |  | 0 | primary | western | intra | digit | PL | 0.070115 |
|  |  | 0 | primary | western | intra | block | PL | 0.213171 |
| Attout & Majerus (2015) | 0.6 | 1 | primary | western | intra | others | UWM | 0.320545 |
|  | 0.6 | 1 | primary | western | intra | others | UWM | 0.447692 |
| Gathercole et al. (2006) | 2.538462 | 1 | primary | western |  | others | PL | 0.351833 |
|  | 2.538462 | 1 | primary | western |  | others | UWM | 0.679201 |
|  | 2.538462 | 1 | primary | western |  | block | VS | 0.259684 |
| Trezise & Reeve (2014) | 0 | 0 | high | western | intra | operation | UWM | 0.4847 |
|  | 0 | 0 | high | western | intra | operation | UWM | 0.693147 |
|  | 0 | 0 | high | western | dressed | operation | UWM | 0.632833 |

*Note*: intra= intra-mathematical problem; dressed= dressed up word problem; UWM= unspecified working memory; CE=central executive; PL=phonological loop; VS=visuo-spatial sketchpad.

a: 0= typically-developing students, 1= partly children with difficulties.
